# A house finch (*Haemorhous mexicanus*) spleen transcriptome reveals intra- and interspecific patterns of gene expression, alternative splicing and genetic diversity in passerines

**DOI:** 10.1186/1471-2164-15-305

**Published:** 2014-04-24

**Authors:** Qu Zhang, Geoffrey E Hill, Scott V Edwards, Niclas Backström

**Affiliations:** 1 Department of Human Evolutionary Biology, Harvard University, 11 Divinity Avenue, Cambridge, MA 02138, USA; 2 Department of Biological Sciences, Auburn University, 331 Funchess Hall, Auburn, AL 36849, USA; 3 Department of Organismic and Evolutionary Biology (OEB), Museum of Comparative Zoology (MCZ), Harvard University, 26 Oxford Street, Cambridge, MA 02138, USA; 4 Current affiliation: Department of Evolutionary Biology, Uppsala University, Norbyvägen 18D, 752 36 Uppsala, Sweden

**Keywords:** House finch, *Mycoplasma gallisepticum*, Gene expression, Transcriptome, Assembly

## Abstract

**Background:**

With its plumage color dimorphism and unique history in North America, including a recent population expansion and an epizootic of *Mycoplasma gallisepticum* (MG)*,* the house finch (*Haemorhous mexicanus*) is a model species for studying sexual selection, plumage coloration and host-parasite interactions. As part of our ongoing efforts to make available genomic resources for this species, here we report a transcriptome assembly derived from genes expressed in spleen.

**Results:**

We characterize transcriptomes from two populations with different histories of demography and disease exposure: a recently founded population in the eastern US that has been exposed to MG for over a decade and a native population from the western range that has never been exposed to MG. We utilize this resource to quantify conservation in gene expression in passerine birds over approximately 50 MY by comparing splenic expression profiles for 9,646 house finch transcripts and those from zebra finch and find that less than half of all genes expressed in spleen in either species are expressed in both species. Comparative gene annotations from several vertebrate species suggest that the house finch transcriptomes contain ~15 genes not yet found in previously sequenced vertebrate genomes. The house finch transcriptomes harbour ~85,000 SNPs, ~20,000 of which are non-synonymous. Although not yet validated by biological or technical replication, we identify a set of genes exhibiting differences between populations in gene expression (*n =* 182; 2% of all transcripts), allele frequencies (76 *F*_*ST*_ ouliers) and alternative splicing as well as genes with several fixed non-synonymous substitutions; this set includes genes with functions related to double-strand break repair and immune response.

**Conclusions:**

The two house finch spleen transcriptome profiles will add to the increasing data on genome and transcriptome sequence information from natural populations. Differences in splenic expression between house finch and zebra finch imply either significant evolutionary turnover of splenic expression patterns or different physiological states of the individuals examined. The transcriptome resource will enhance the potential to annotate an eventual house finch genome, and the set of gene-based high-quality SNPs will help clarify the genetic underpinnings of host-pathogen interactions and sexual selection.

## Background

Understanding the hereditary components underlying trait variation in natural populations is key to answering a range of fundamental questions in evolutionary biology, but advancing basic insight into evolutionary relevant genotype-phenotype interactions is not trivial. Two essentials of this endeavor are quantification of phenotypic variation in traits that influence individual fitness in natural settings [1] and collection of information on DNA sequences, linkage maps, gene expression profiles or other genomic resources spanning the genome of the focal organism [eg. [2,3]. The recent progress in data collection in evolutionary genetics research, mediated predominantly by advancements in DNA sequencing, allows one to rapidly generate vast amounts of genomic data at reasonable cost, even for organisms that are genetically poorly known [4]. These advances facilitate detailed analyses of DNA sequence evolution in almost any species of interest, whether to scan for signs of positive selection, characterize genome-wide divergence between lineages or identify associations between genomic regions and phenotypic traits ([5,6], The Heliconius Genome Sequence Consortium, [7]). Consequently, the main limiting factor for developing model systems for evolutionary genomics is acquisition not of genomic resources but rather of sufficient evolutionarily relevant phenotypic and fitness data. The implications of this trend are that taxa for which long-term ecological data have already been collected (ecological model species) will be at the forefront of evolutionary genomics research on natural populations [1].

A long-term model species for studying sexual selection and host-pathogen interactions in the wild is the house finch (*Haemorhous mexicanus*) [8]. The species is native to western North America [9], but shipping of native birds from the western US to pet traders in the east resulted in the establishment of a feral house finch population in the New York City area around 1940 [10]. Over the subsequent decades, this eastern population of house finches expanded its distribution across half of the continent and the population size increased exponentially. The house finch became one of the most common bird species in the eastern US and census estimates suggested a population size in the range of hundreds of millions [11]. As a result of a *Mycoplasma gallisepticum* (MG) epizootic, which emerged in the Washington D.C. area in 1994 and subsequently spread rapidly over the eastern range [12,13], populations of house finches in the eastern US and eastern Canada declined by approximately 50% between 1994 and 1997 [14]. After the initial precipitous decline, the population remained stable and there were indications of increasing MG resistance in eastern populations [15-17]. The unique demographic history of the house finch, distinct and evolutionary important carotenoid-based color variation between males [11,18], and the selection regime brought about by the MG epizootic have made it a model species for many issues in natural selection, sexual selection, and evolutionary genomics, including morphological evolution in response to regional climate [19,20] plumage coloration and its role in sexual selection [18,21,22], gene expression evolution [17,23-25], the genomic effects of founder events [26-28], and patterns of molecular evolution in candidate genes [29,30], and across the genome [31]. These are merely a handful of examples of the substantial knowledge about phenotypes of relevance to evolutionary biology that is represented in this study system [8].

Here we present high coverage spleen transcriptomes from two populations of the house finch, one representing birds from a population in Arizona (AZ) that has never been exposed to MG and the other representing birds from a population in Alabama (AL) that had been exposed to MG for 15 years at the time of collection. So far, genomic comparisons between these two populations have included expression profiling based on macro- and micro-arrays [17,23,32] and small-scale, partial candidate gene sequences [25] or anonymous genetic marker data [26]. The transcriptome assembly provided here is a novel resource for forthcoming comparative and functional studies within house finches and among birds in general. Briefly, we describe our sequencing and assembly procedure and the results that we obtained using the zebra finch as a resource for comparative study of gene expression evolution in avian spleen. We follow up with functional annotation and analysis of genetic differentiation in gene expression between the two focal house finch populations studied here to identify genes exhibiting extensive differentiation between these populations. Such differentially expressed genes are candidate genes for MG resistance, and these genes will be important targets for subsequent detailed functional analyses to understand host-pathogen interactions in this system and in general. A preliminary report of this transcriptome (*n* = 4,398 genes), pooled across both populations, helped to clarify long-term patterns of protein-evolution in birds and other amniotes [31]. The present study focuses on a transcript set over twice as large (*n* = 9,646 genes) and on expression differentiation between house finch populations and between house finch and zebra finch, the only other passerine bird for which spleen expression data is available.

## Results

**Figure 1:**
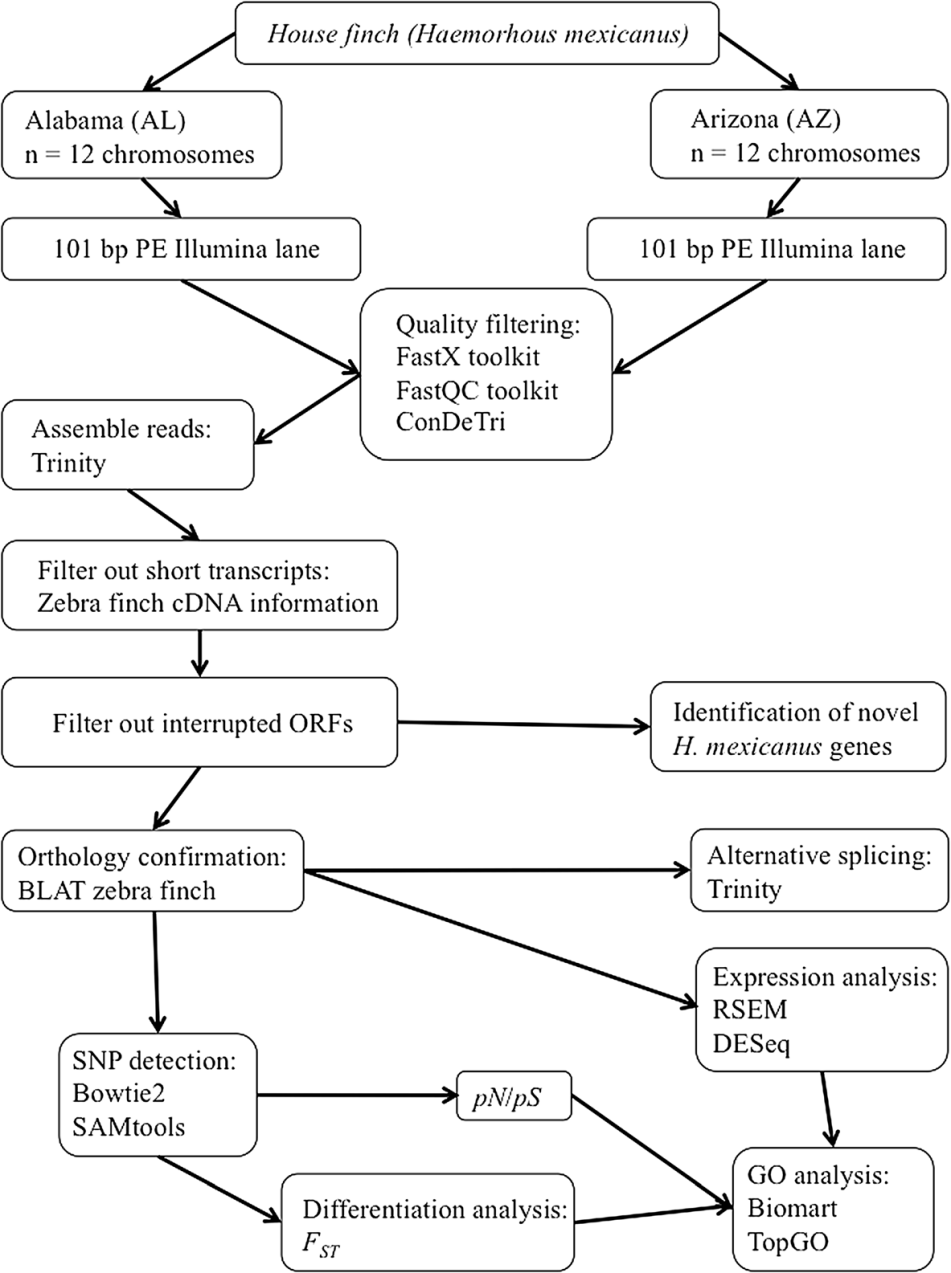
Schematic work-flow illustrating the main steps from the sampling to the final analyses.

The Illumina HiSeq run (1 lane per library) generated in total 251.1 million reads (25.4 Gb) for the AL population and 250.9 million reads (25.3 Gb) for the AZ population. After quality trimming (threshold = phred score > 25), 145.0 million and 147.1 million reads with paired-end information and 36.4 and 36.0 million reads without pairing information (in total 181.4 and 183.0 million reads, corresponding to, respectively, 15.9 and 16.0 Gb) remained for the AL and the AZ populations, respectively (see Figure 1 for work-flow and Additional file 1: Table S1 for details).

### Summary of the transcriptome assembly

**Figure 2:**
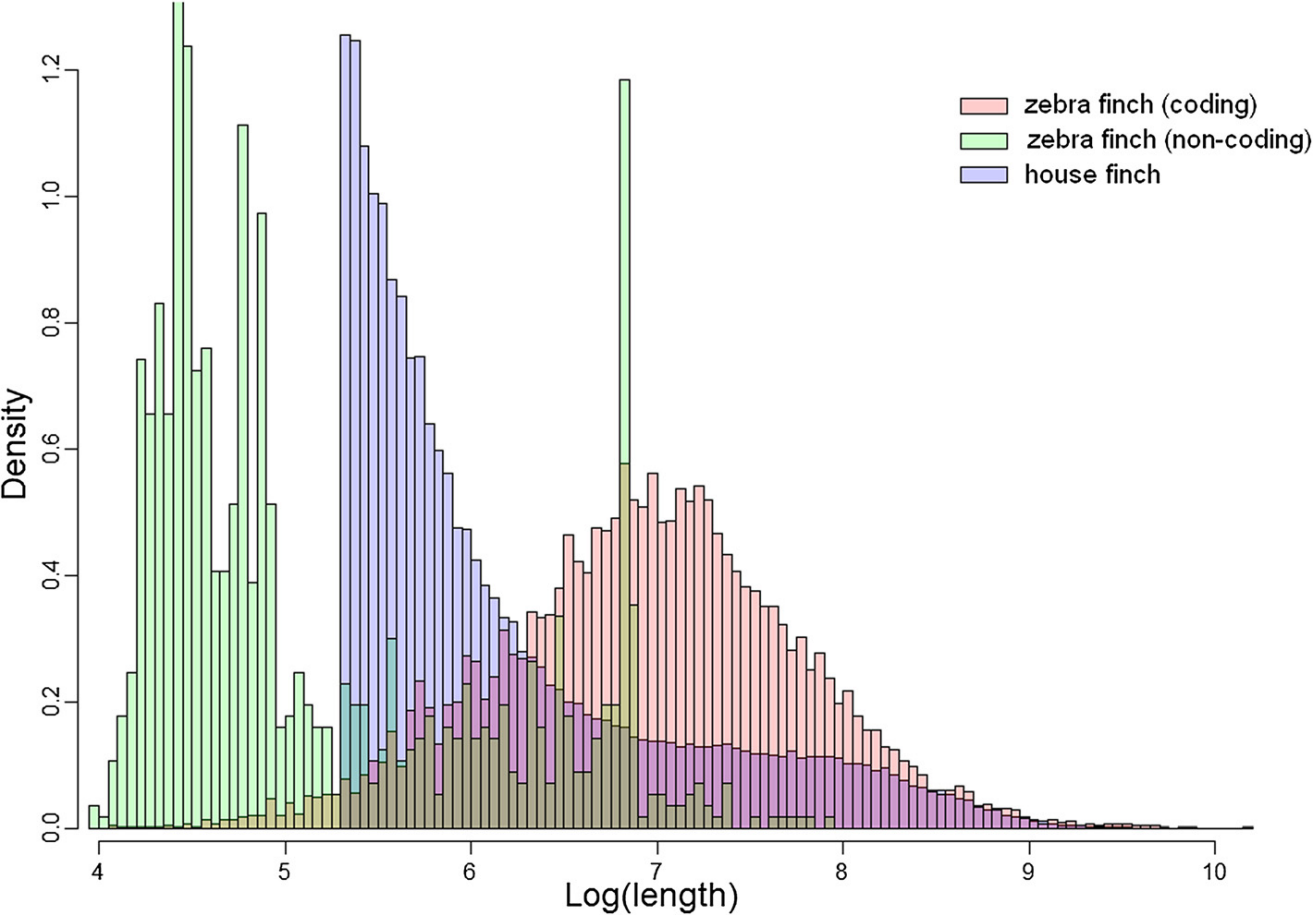
**Distribution of zebra finch coding (pink bars) and non-coding (green bars) transcript lengths and house finch transcript lengths (blue bars) after applying the cut-off threshold of 462 bp for including a house finch transcript in the data set.** In general, non-coding transcripts are shorter than coding transcript, but we also observe a spike around 1000 bp in the zebra finch non-coding transcripts, which may represent long non-coding RNAs.

Quality-trimmed Illumina reads (*n* = 364.4 million) were used to assemble the house finch transcriptome. Using Trinity*,* we obtained 222,678 reconstructed transcripts with a mean size of 827 bp and a median size of 347 bp. The shorter transcripts, which likely contain substantial numbers of non-coding RNAs, were filtered out using our size cut-off threshold (see Methods). Using this cutoff on zebra finch transcripts, 98.5% (15,302/15,542) of all zebra finch cDNAs encode proteins (Figure 2). This high incidence of protein-encoding cDNAs indicates that the procedure to filter out non-coding house finch cDNAs was likely stringent. When applying this filter to assembled house finch transcripts, we retained 82,384 transcripts, or 37% of the initial set. We estimated the size of intact open reading frames (ORFs) for each of these qualified transcripts and discarded those with an ORF ≤ 300 bp. This resulted in 47,542 retained transcripts, which we designate as the unfiltered set. Finally, we used BLAT [33] to align the retained transcripts to coding cDNAs in the zebra finch and filtered out transcripts using criteria mentioned in Methods, yielding a high-quality transcript set of 9,646 house finch coding cDNAs with orthologs in zebra finch. We define this set as the filtered set and used it as the primary working set for subsequent expression and comparative sequence analyses (Additional file 1: Table S2).

### Different splenic expression patterns between house finch and zebra finch

**Figure 3:**
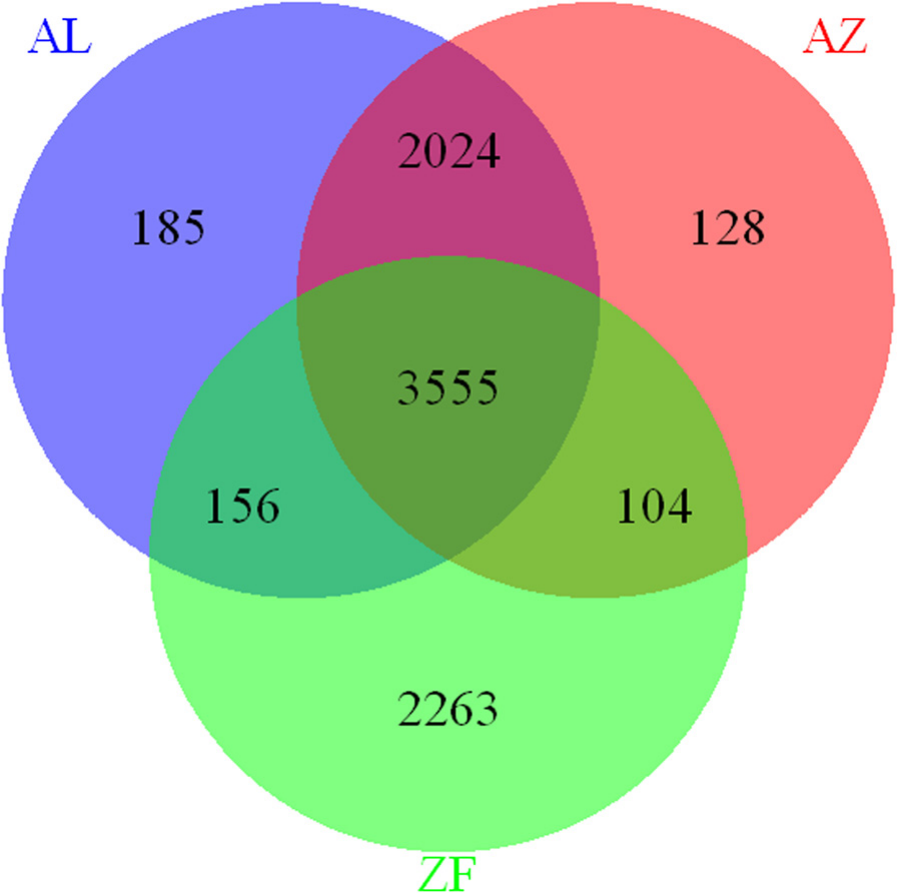
**A Venn diagram illustrating the number of genes expressed in spleen in zebra finch (green) and in the Alabama (historically exposed, blue) and Arizona (historically unexposed, red) house finch populations.** Overlapping areas between population distributions indicate the number of genes expressed in common between involved populations.

We compared the splenic expression profiles in the two house finch populations to each other and to previously published data from the zebra finch [34]. A transcript was defined as expressed if it has at least one CPM (read count per million total reads). Among the 11,769 zebra finch transcripts identified that could be mapped to the Ensembl 69 zebra finch assembly (the version used in our study, see above), a total of 8,415 transcripts were expressed in at least one tissue and a subset of 6,078 transcripts were expressed in the zebra finch spleen. The corresponding value for the house finch was 6,152 (5,920 in the AL population and 5,811 in the AZ population, respectively; Figure 3). 3,555 transcripts were expressed in all three groups, 2,263 were uniquely expressed in zebra finch, 2,024 were uniquely expressed in house finch, and 185 and 128 transcripts were uniquely expressed in AL and AZ populations, respectively (Figure 3). Different GO terms were enriched in different groups (for details see Additional file 1: Tables S3 and S4); in general, genes related to protein binding were uniquely expressed in house finch spleen and genes related to oxidase activity were uniquely expressed in zebra finch spleen (Additional file 1: Table S3), whereas genes related to metabolism were uniquely expressed in one or the other of the house finch populations (Additional file 1: Table S4).

### Novel genes in the house finch

A question of interest was to identify potential novel (unique) protein-coding genes that might have evolved unique functions in the house finch or related lineages. To investigate this, we first used the CPC program [35] to predict the coding potential for each of the transcripts assembled *de novo* in the house finch. Out of the initial 222,678 reconstructed transcripts, 33,767 (15%) were predicted as coding and these included as many as 27,241 (81%) transcripts that had no identifiable zebra finch ortholog. These 27,241 transcripts were mapped against all known chicken, *Anolis* and human coding cDNAs, and we identified an additional 617 transcripts with known chicken orthologs, 46 with *Anolis* orthologs and 13 with human orthologs. Among the remaining 26,565 transcripts, we identified house finch genes not yet found in birds by first calculating the median coding potential score (6.39) and the median size (2,604 bp) of the 7,202 transcripts with an identified ortholog and compared these values to the set of genes with no identified ortholog in any of the other four species. This analysis revealed that 4,940 (18.6%) of the 26,565 transcripts were longer and had larger coding potential than the median values of the known coding transcripts. Next, we calculated the median expression level of known coding genes (3.9 and 3.7 TPM (transcripts per million total reads) for AL and AZ respectively), and found that 511 and 502 of the novel transcripts in AL and AZ, respectively, had higher expression than the median. Of them, 436 (AL) and 425 (AZ) were highly similar isoforms of transcripts with known orthologs, i.e. false positives generated through the *de novo* assembly process. Excluding these we had 75 (AL) and 77 (AZ) transcripts of which 20 and 19 partially overlapped (≥80% identity but <50% query length coverage) known zebra finch protein-coding genes. All the transcripts that were identified as potential orthologs in the latter steps had low query length coverage and that is likely the reason why we failed to detect them in the initial BLAST search. This could possibly be explained by partial duplications of the orthologous genes or substantial differences in splicing isoforms between the two species. The remaining 55 (AL) and 58 (AZ) transcripts that could not be mapped to cDNAs from zebra finch were further aligned to the zebra finch genomic sequence. For certain transcripts that were different splice forms of the same genes, only the longest splicing form was used in the alignment. This resulted in significant alignment scores for 45 query transcripts in each population. Of these, 31 (69%) and 32 (71%) were aligned at various length coverage with > =80% sequence similarity and exhibited clear exon-intron structures in zebra finch, implying that these are actually functional genes with incomplete annotation in the zebra finch. 14 and 13 transcripts (12 in common, 15 transcripts in total) were not aligned at all and these constitute a set of novel genes in the lineage leading to house finch at some point after the split from zebra finch.

### Expression differences between historically exposed and unexposed house finch populations

**Figure 4:**
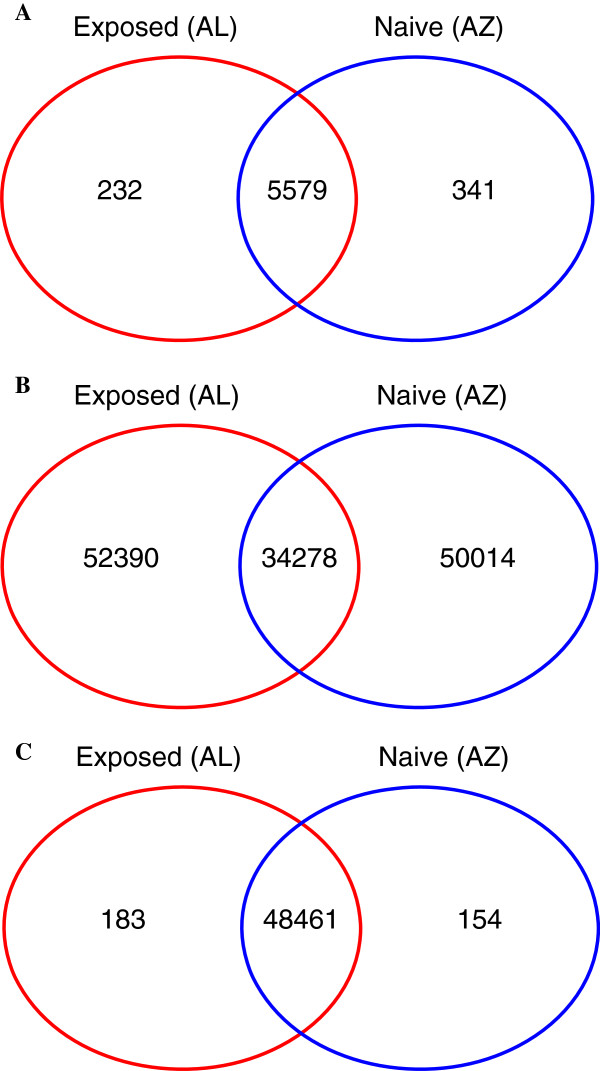
Venn diagrams illustrating the number of genes expressed uniquely in a single population and genes with shared expression between populations (A), the number of identified high-quality shared and private SNPs (B) and unique and shared splice variants (C) between the exposed (AL) and naïve (AZ) house finch populations.

We compared the expression profiles of transcripts in the filtered set in the two house finch populations: AL (historically exposed to MG) and AZ (historically unexposed). Expression levels were estimated using the RSEM package [36], and expression differences were assessed using DESeq [37]. Using the topGO package in the Bioconductor project frame [38] and applying a false discovery rate (FDR) of 0.05 and a minimum fold change of at least two in either direction for the 8,981 transcripts with noticeable expression (>1 CPM) we found that 182 (~2%) transcripts were differentially expressed between the house finch populations (Figure 4, Additional file 1: Table S4). A subsequent functional enrichment analysis of the these transcripts showed overrepresentation of a total of 22 terms, 14 related to biological processes, one related to cellular component and seven related to molecular functions (Table 1).

**Table 1:** Gene ontology terms for genes differentially expressed between the two house finch populations when comparing expression profiles of the spleen between populations

**GO term**		**Annotated**	**Significant**	**Expected**	**Classic p**	**Corrected p**
GO:0044281 (BP)	Small molecule metabolic process	818	39	17	3.7*10^−7^	3.0*10^−3^
GO:0019752 (BP)	Carboxylic acid metabolic process	284	20	6	1.7*10^−6^	3.0*10^−3^
GO:0043436 (BP)	Oxoacid metabolic process	284	20	6	1.7*10^−6^	3.0*10^−3^
GO:0006082 (BP)	Organic acid metabolic process	285	20	6	1.8*10^−6^	3.0*10^−3^
GO:0042180 (BP)	Cellular ketone metabolic process	296	20	6	3.2*10^−6^	5.0*10^−3^
GO:0006520 (BP)	Cellular amino acid metabolic process	158	14	3	5.2*10^−6^	6.0*10^−3^
GO:0006725 (BP)	Cellular aromatic compound metabolic process	57	8	1	2.3*10^−5^	2.4*10^−2^
GO:0009156 (BP)	Ribonucleoside monophosphate biosynthetic process	18	5	0	2.7*10^−5^	2.5*10^−2^
GO:0009124 (BP)	Nucleoside monophosphate biosynthetic process	19	5	0	3.6*10^−5^	2.9*10^−2^
GO:0072522 (BP)	Purine-containing compound biosynthetic process	66	8	1	6.7*10^−5^	4.7*10^−2^
GO:0009161 (BP)	Ribonucleoside monophosphate metabolic process	22	5	0	7.8*10^−5^	4.7*10^−2^
GO:0009127 (BP)	Purine nucleoside biosynthetic process	12	4	0	8.4*10^−5^	4.7*10^−2^
GO:0009168 (BP)	Purine ribonucleoside biosynthetic process	12	4	0	8.4*10^−5^	4.7*10^−2^
GO:0006563 (BP)	L-serine metabolic process	5	3	0	9.0*10^−5^	4.7*10^−2^
GO:0009112 (BP)	Nucleobase metabolic process	13	4	0	1.2*10^−4^	5.5*10^−2^
GO:0009123 (BP)	Nucleoside monophosphate metabolic process	24	5	1	1.2*10^−4^	5.5*10^−2^
GO:0034654 (BP)	Compound biosynthetic process	94	9	2	1.5*10^−4^	6.4*10^−2^
GO:0009113 (BP)	Purine nucleobase biosynthetic process	6	3	0	1.8*10^−4^	7.3*10^−2^
GO:0043292 (CC)	Contractile fiber	41	7	1	6.0*10^−6^	6.0*10^−3^
GO:0019842 (MF)	Vitamin binding	66	8	1	7.9*10^−5^	4.2*10^−2^
GO:0016742 (MF)	Transferase activity	5	3	0	9.6*10^−5^	4.2*10^−2^
GO:0016840 (MF)	Carbon-nitrogen lyase activity	5	3	0	9.6*10^−5^	4.2*10^−2^
GO:0016741 (MF)	Transferase activity, transferring one-carbon groups	108	10	2	1.0*10^−4^	4.2*10^−2^
GO:0003824 (MF)	Catalytic activity	2828	84	61	1.2*10^−4^	4.2*10^−2^
GO:0030170 (MF)	Pyridoxal phosphate binding	37	6	1	1.2*10^−4^	4.2*10^−2^
GO:0070279 (MF)	Vitamin B6 binding	37	6	1	1.2*10^−4^	4.2*10^−2^
GO:0016712 (MF)	Oxidoreductase activity	6	3	0	1.9*10^−4^	4.6*10^−2^
GO:0016740 (MF)	Transferase activity	959	37	21	2.4*10^−4^	4.8*10^−2^

*MF*, Molecular function; *CC*, Cellular component. GO term is the identification number for the gene ontology term and given is also the annotated, significant and expected number of genes for each term and the uncorrected (Classic p) and corrected (Corrected p) p-values.

### SNP frequencies and nucleotide diversity in the house finch transcriptome

**Figure 5:**
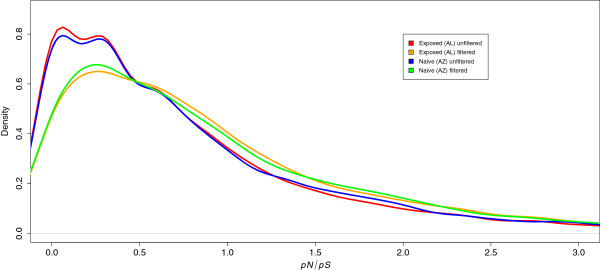
**Density distribution plots for the estimates of *p***_***N***_ ***/p***_***S***_**in the two house finch populations for the two datasets; 1) all identified SNPs (n ≈ 190,000 SNPs per population, red (AL,**$\bar{x}\pm \sqrt[{2}]{\mathrm{VAR}}$**= 0.83 ± 0.98) and blue (AZ, 0.86 ± 0.99) curves) and, 2) quality filtered SNPs (n ≈ 85,000 SNPs per population, orange (AL, 1.01 ± 1.04) and green (AZ, 1.00 ± 1.05) curves).**

**Figure 6:**
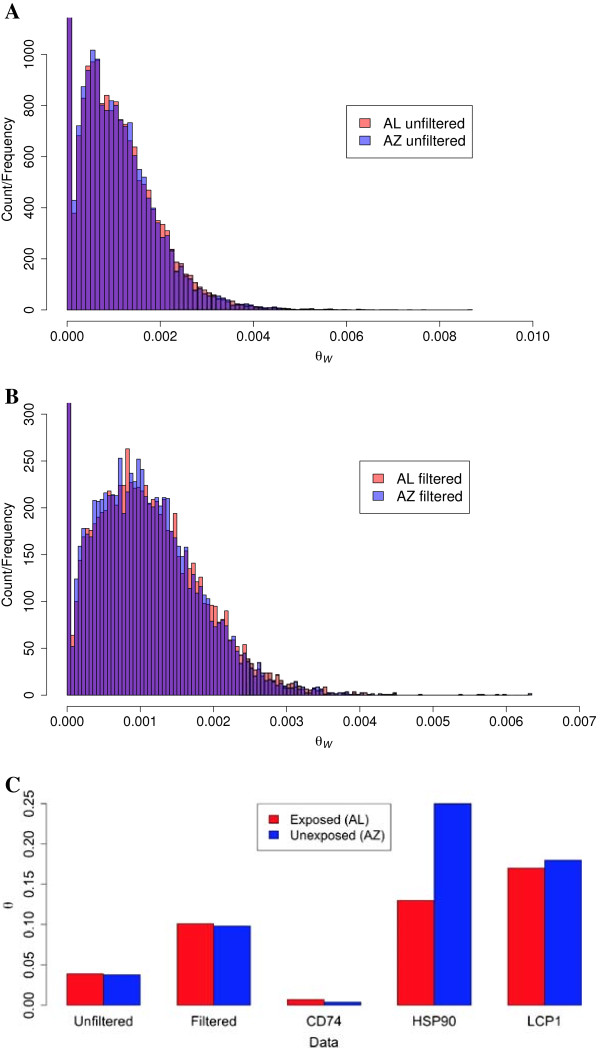
**Histogram illustrating the distribution of *θ***_***W***_**– values for transcripts using the unfiltered (A) and filtered (B) data sets. Red bars show the values for AL and blue bars show the distribution of values for AZ.** The y-axis has been cut at 1,000 **(A)** and 300 **(B)** to get a clearer picture of the distribution and the overlap (purple) between populations. For the unfiltered data set the number of transcripts with *θ*_*W*_ = 0 was 1,784 for AL and 1,775 for AZ and the corresponding values for the filtered data set were 32,165 (AL) and 32,183 (AZ). **Panel C** shows the average diversity estimates (x100) for the data from this study (unfiltered and filtered data, *θ*_*W*_) and *θ* estimates (π) from a re-sequencing effort of upstream regulatory sequences of the three genes *CD74*, *HSP90* and *LCP1*[25] for exposed (AL, red bars) and unexposed (AZ, blue bars) populations.

Using the criteria outlined in Materials and Methods, we identified a total of 193,037 and 187,253 SNPs in the unfiltered set in the AL and AZ populations, respectively. 86,668 (AL) and 84,292 (AZ) SNPs remained in the filtered data set of 9,646 house finch coding transcripts with orthologs in zebra finch, and of these, 34,278 SNPs were shared between populations (Figure 4). We classified the SNPs as either non-coding (*n*_*AL*_ = 43,297; *n*_*AZ*_ = 41,502), synonymous (*n*_*AL*_ = 21,740; *n*_*AZ*_ = 21,537) or non-synonymous (*n*_*AL*_ = 21,740; *n*_*AZ*_ = 21,253) and calculated the *p*_*N*_/*p*_*S*_ ratio for each transcript for both the unfiltered and the filtered polymorphism datasets. The frequency distributions of *p*_*N*_/*p*_*S*_ in the unfiltered and filtered SNP data sets are presented in Figure 4. The filtered set showed a larger proportion of transcripts with a higher *p*_*N*_/*p*_*S*_ (Figure 5). We used the method of Watterson [39] to estimate global and transcript specific diversity estimates (*θ*_*W*_). The AL population had slightly higher diversity estimates (*θ*_*W*_ unfiltered data: 3.89*10^−4^ ± 7.22*10^−4^; *θ*_*W*_ filtered data: 1.01*10^−3^ ± 0.78*10^−3^) than the AZ population (*θ*_*W*_ unfiltered data: 3.78*10^−4^ ± 0.71*10^−4^; *θ*_*W*_ filtered data: 0.98*10^−5^ ± 0.77*10^−3^) although the difference between populations was not statistically significant for either the filtered (Wilcoxon’s Test, W = 47,180,752, p-value = 0.088) or the unfiltered (W = 1,132,672,156, p-value = 0.468) data sets (Figure 6). Comparison of these estimates of diversity with similar estimates from house finches recently made for other sequence-based markers, including cis-regulatory regions of candidate genes for resistance [21], shows that house finch transcript diversity is in the range discovered for other markers (Figure 6).

### Genetic differentiation between AL and AZ populations

**Figure 7:**
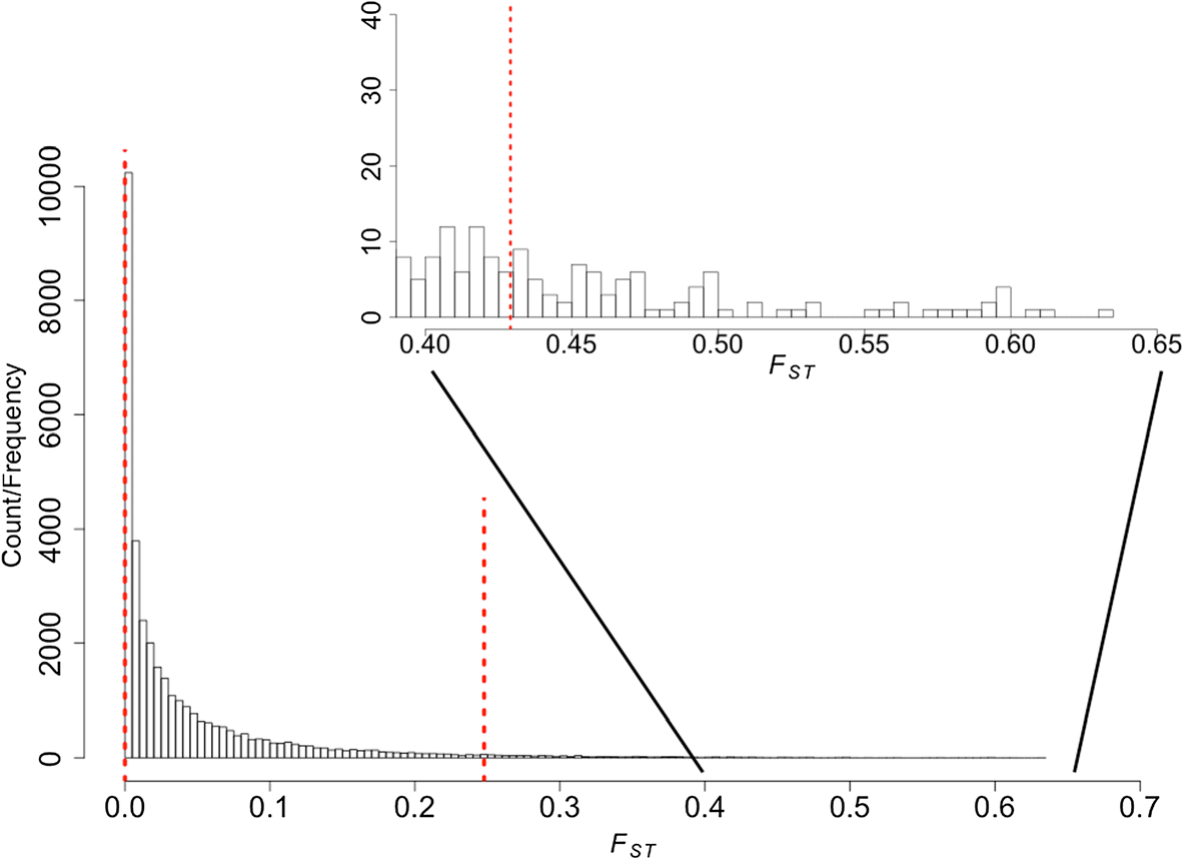
**Histogram illustrating the distribution of *F***_***ST***_**values between the two populations calculated for all transcripts containing at least 3 high quality SNPs.** The vertical, dotted red lines indicate the 2.5% (left, main figure), 97.5% (right, main figure) quantiles and the threshold value for > 3 standard deviations away from the arithmetic mean (insert showing distribution of *F*_*ST*_ values > 0.3).

To estimate the degree of population differentiation from our data we calculated the *F*_*ST*_ statistic. We initially used the 34,278 SNPs shared between the AL and the AZ populations. These SNPs mapped to 6,746 coding transcripts in the filtered set. Among these we selected 4,474 transcripts that contained at least three SNPs and calculated *F*_*ST*_. We found that most transcripts had a low *F*_*ST*_ (mean = 0.0443, Figure 7) and only 76 (~1.7%) transcripts showed *F*_*ST*_ – values at least 3 standard deviations above the mean value (Figure 7). In this set of 76 transcripts with unusually high values of *F*_*ST,*_ we did not detect any enriched functional GO terms.

### Fixed differences between AL and AZ populations

We identified a set of high-quality private SNPs and potential fixed differences by applying a series of strict filters. Using SAMtools, we first identified SNPs with non-reference allele fixed in one population and reference allele fixed in the other population and present with a read coverage >3 in both populations; variants within 5-bp were discarded to avoid possible false positives due to read misalignment. This resulted in a set of 806 (AL) and 640 (AZ) private variants in the unfiltered set; 317 (AL) and 274 (AZ) of these were classified as non-synonymous polymorphisms. 235 (AL) and 201 (AZ) of the private polymorphisms were retained in the filtered set, and of these, 79 and 81 were non-synonymous changes present in 74 proteins in AL and AZ, respectively (Additional file 1: Table S5). We then looked for cases where at least two fixed non-synonymous differences were present in a single transcript, as this could indicate recent strong directional selection. Three of the five genes that possessed multiple fixed differences had Ensembl identification numbers in the zebra finch gene set and they are listed in Table 2. Briefly, the list of genes contained two genes with unknown function, one gene associated to the double-strand break repair mechanism and two genes involved in disease response; a heat-shock associated (*HSPBAP1*) and a T-cell precursor (*THYMIS*) gene (Table 2).

**Table 2:** Five genes with Ensembl entries available in the zebra finch containing at least two fixed non-synonymous differences between Alabama (AL, exposed) and Arizona (AZ, naïve) house finch populations

**Ensembl transcript ID**	**Gene name**	**Function**
ENSTGUT00000004575	HSPBAP1*	Heat-shock PB associated protein 1
ENSTGUT00000011244	*LIG4*^*†*^	Ligase IV, DNA, ATP-dependent
ENSTGUT00000006730	Novel gene	Unknown
ENSTGUT00000012246	THYMIS*	Thymocyte selection associated
ENSTGUT00000016683	Novel gene	Unknown

Two genes have unknown function in birds, one is related to double-strand break repair (†) and two are related to immune response (*).

### Patterns of alternative splicing

**Figure 8:**
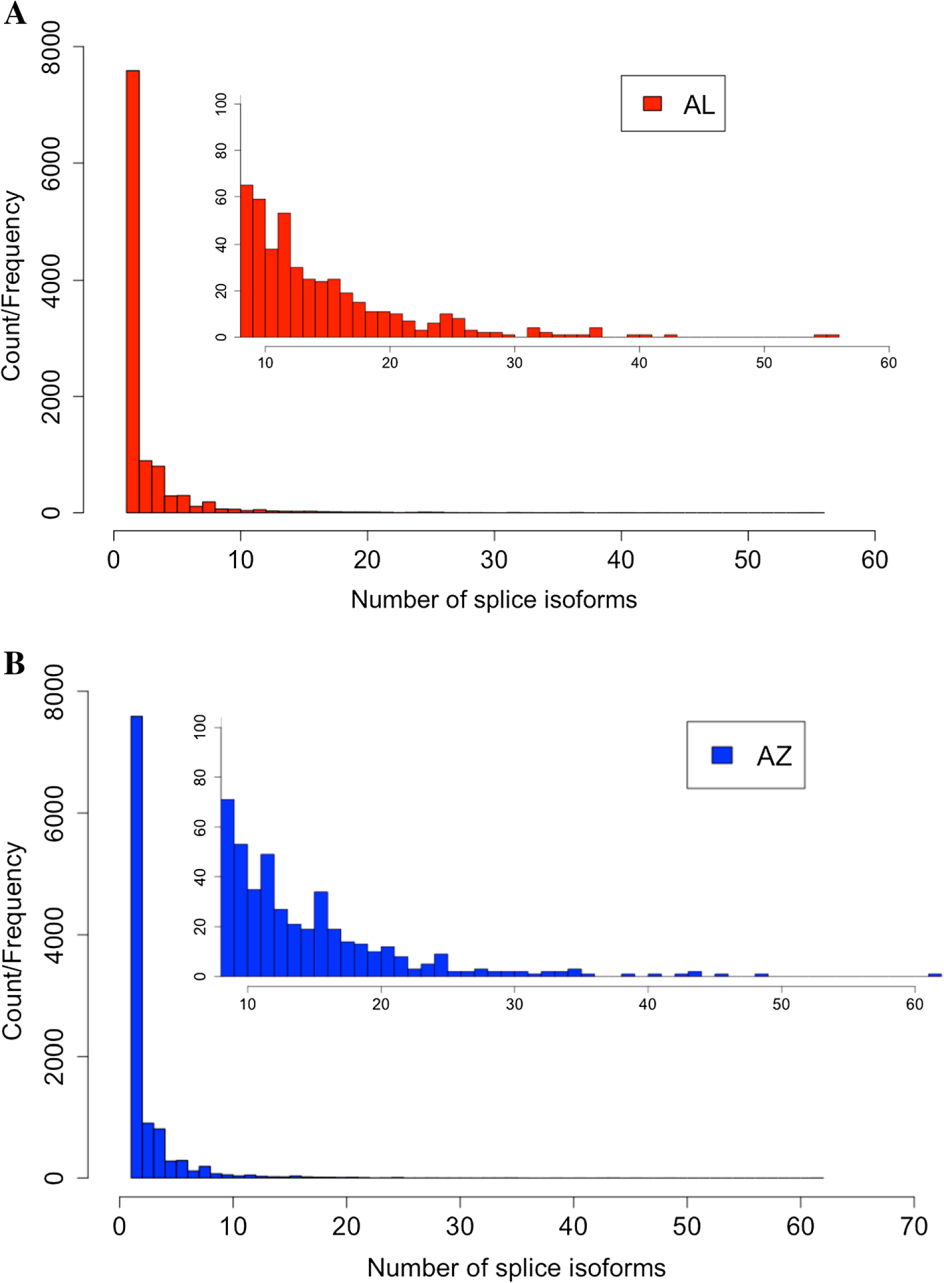
**Histogram illustrating the distribution of the number of splice variants per transcript within the AL (panel A, red bars) and AZ (panel B, blue bars) population, respectively.** The insert on each panel shows the distribution for genes with the number of splice variants > 10.

Finally, we investigated patterns of alternative splicing in our house finch populations. Of the 47,542 house finch transcripts in the unfiltered set with an ORF longer than 300 bp that mapped to zebra finch cDNAs, 17,652 could be retained when requiring a single gene alignment with at least 90% similarity between species and detectable at more than one CPM in either population. Altogether, these transcripts encompassed 9,167 and 9,007 annotated zebra finch cDNAs in the AL and AZ populations, respectively. The median number of splicing forms in these cDNAs was two in both the AL and the AZ population, and there were 5,333 (58.2%) and 5,332 (59.2%) cDNAs with only one splice variant in the AL and AZ population, respectively. 3,834 (AL) and 3,675 (AZ) cDNAs had two or more splice variants; with a range of 2-23 (AL) and 2-22 (AZ) variants (Figure 8). Of all variants called, 183 (1% of all splice variants corresponding to 154 different cDNAs) and 164 (0.9% of all splice variants corresponding to 131 different cDNAs) transcripts were uniquely found in the AL and the AZ population, i.e., with more than one CPM in one population and no detectable expression in the other population, respectively (Figure 4).

## Discussion

Transcriptomes are a valuable genomic resource for species of ecological and evolutionary significance because they contain likely targets of natural selection and can provide a catalog of protein-coding regions of interest. Transcriptomes are likely enriched for targets of natural and sexual selection not only because the genes themselves may harbor mutations influencing relevant phenotypes but also because regulatory sequences are enriched in the vicinity of coding regions [40]; hence any signals of selection on a regulatory mutation or associations with phenotypic variation may be detectable in genetic variants in nearby coding regions. Here we report a draft spleen transcriptome assembly of the house finch, a species of importance for sexual selection and host-pathogen interactions in the wild [8,17,18,23,25,27,31,41]. Besides being one of the first non-normalized RNA sequencing efforts available for non-model avian taxa [42-44], our transcriptome is an important resource for comparative genomics studies within birds [35] and for detailed analyses of the genetic basis of pathogen resistance in this particular system. Because we sampled only two populations, our sampling design does not allow us to distinguish many interlocking events that could shape patterns of expression and polymorphism in these birds, including the introduction and adaptation to a novel environment in the eastern US and the effect of the MG epizootic on levels of polymorphism. Still, after stringent quality filtering using information from zebra finch and chicken, the best-annotated avian genome assemblies, we can state that the core set of transcribed house finch sequences comprises almost 10,000 unique genes. This set of genes is an invaluable resource for forthcoming efforts aimed at pinpointing the genetic basis of evolutionarily important traits in the house finch, such as resistance to *Mycoplasma gallisepticum*[17,24,25,31] and the intensity of plumage redness in males [8,18,21,22].

### Expression differences between species

When comparing the set of genes expressed in the house finch spleen to a comparable set of genes expressed in the zebra finch spleen [34] we found that only 3,555 of the total set of 8,415 genes were expressed in both species. The spleen is a relatively small organ in birds and, although thorough studies of spleen function are uncommon, it plays an obvious role in the universal immune response [45]. It was therefore somewhat surprising to find that a large proportion of the entire coding gene set for zebra finches (*n* = 17,488 genes) is expressed in the spleen and that such a large proportion of these genes are uniquely expressed in one species and enriched for different gene ontology terms. By contrast, only 185 and 128 transcripts were uniquely expressed in one of the two house finch populations studied here. However, most of these genes that were differentially expressed between house finch populations have an average level of expression that is low compared with genes that shared expression between populations (mean TPM of 0.41 versus 0.46 in AL and 0.36 versus 0.42 in AZ). This low level of expression overlap, combined with our small sample sizes per population, means that the probability of failing to detect such genes in one population or the other is high. These factors make the expression differences that we observed between zebra finch and house finch spleen even more striking. In addition to evolutionary divergence in gene expression, undoubtedly many of the differences found between zebra finch and house finch spleen are attributable to overall methodology and the physiological state of the individual birds used for RNA-seq.

### Novel genes in the house finch transcriptome

Of particular value to the community of researchers studying house finches is the establishment of a catalog of transcripts and genes, including those that are not previously characterized in other organisms. Therefore, we attempted to identify transcripts with high coding potential that had not yet been found in birds. By using a strict set of filters and information about coding potential and reading frames from zebra finch, we identified 15 genes that were uniquely present in the house finch transcriptome data set compared to other birds. These genes might be genes that evolved novel function in the house finch lineage after divergence from the zebra finch around 50 million years ago [46] or they may represent ancestral genes that lost function in chicken and zebra finch and rapidly accumulated mutations so that they no longer can be identified as potential homologs in standard reciprocal BLAST analyses. This set of novel genes lacking orthologs in zebra finch or chicken should be primary targets in subsequent analyses aimed at characterizing the genetic basis of novel functions in the lineage leading to the house finch. Increased taxon sampling, which presumably will find these genes in additional relatives of house finches, will increase the power of our inferences about novel genes and their functions.

### Expression differences between populations

In addition to the small set of genes uniquely expressed in one of the two populations (see above), we also found significant expression-level differences for a set of genes when comparing the historically exposed (AL) and unexposed (AZ) population of house finches. Using a strict filtering pipeline we collected a high-quality set of 8,981 genes with CPM >1, 182 (~2%) of which were significantly differentially expressed between house finch populations. This gene set was enriched for genes related to metabolic processes, vitamin binding, transferase- and catalytic activity. Because the sampling was not designed to make explicit conclusions about globally different patterns of gene expression in the spleen between these two populations - for example by keeping all birds in a common environment for a considerable time period before sampling – the overrepresentation of metabolic processes likely partly reflects differences in the home environment for the different populations. Differential expression of metabolic genes could, for example, be in part due to a difference in ambient temperature at the time of sampling in Arizona as compared to Alabama [47]. It should also be stressed that biological replicates and/or validation of gene expression differences with alternative methods will be needed to verify differential expression patterns between the house finch populations.

### Genetic variation and rates of non-synonymous substitution

Using a series of stringent filtering criteria we identified a set of high-quality SNPs in the transcriptome sequence of the house finch, allowing us to estimate the SNP density for the transcribed part of the genome and to assess within- and between-population genetic variation. Overall, we identified roughly 85,000 high-quality SNPs in each of the populations. The average SNP density was hence very similar between populations; in fact, although not significant (p-value = 0.2941, Fisher’s exact test) after correcting for the number of expressed nucleotides (22,950,549 and 22,434,784), the number of SNPs discovered in the recently founded AL population (*n =* 86,668) was slightly higher than in the native AZ population (*n =* 84,292). This pattern is consistent with the idea that neither the artificial introduction of house finches into eastern North America nor the *Mycoplasma* epizootic has dramatically reduced levels of heterozygosity in coding regions in the eastern population. Population genetic considerations suggest that the estimates for each population are unlikely to be strongly affected by our relatively small sample size (*n* = 12 chromosomes per population), which is large enough to capture most variation [48,49], especially given that these populations are not strongly structured [26]. Sample sizes are also unlikely to explain the pattern because our estimates of genetic diversity were if anything slightly larger for the eastern than for the western US, and the error in these estimates is influenced much more so by the number of loci than the number of individuals. The putative bottleneck in the eastern house finch population was short and the population has been rapidly growing for the last 60 years, perhaps resulting in lower levels of drift and a higher incidence of polymorphisms, albeit at low frequency. Additionally, although the selection event as a result of the *Mycoplasma* epizootic may not have been strong, resulting in a negligible effect on levels of polymorphism, any explanation must also reconcile the fact that several surveys of genetic variation in pre- and post- epizootic finches have found significantly lower levels of variation in the east than in the west. Such studies include analyses of mitochondrial DNA, microsatellites and MHC genes [27,41]. Aside from the mtDNA and MHC results, which are likely to be idiosyncratic due to issues of linkage, ploidy and balancing selection, the contrasting patterns in these studies and our results suggest that the differences may lie largely in differences in evolutionary dynamics of coding versus noncoding (microsatellite) loci [50-52]. Hawley *et al.*[27] found for example increased diversity in MHC class II genes in post-epizootic birds from eastern United States, potentially reflecting disease mediated balancing selection.

We observed that only 40% of the SNPs were shared between populations. Given the short divergence time and the high level of heterozygosity in the introduced population, the fraction of shared polymorphisms is expected to be high; hence, the lower-than-expected level of shared polymorphism observed here is likely a result of relatively small sample sizes from each range resulting in that many, especially low-frequency, polymorphisms that are indeed segregating in both populations are detected in only one of the populations. As a complementary test to assess if the introduced AL population might have been affected by either the demographic history or the *Mycoplasma* epizootic (or both), we looked at the *p*_*N*_/*p*_*S*_ ratios calculated for each population separately using both the unfiltered and the filtered set of SNPs. The underlying hypothesis was that the historically exposed and potentially bottlenecked AL population should show a higher ratio of non-synonymous to synonymous polymorphisms as a consequence of less efficient selection against slightly deleterious non-synonymous alleles drifting to higher frequency and hence more easily detectable in our sample. In neither of the two data sets did we observe a difference in *p*_*N*_/*p*_*S*_ between the native AZ population and the introduced AL population, again suggesting that neither the introduction itself nor the exposure to the epizootic have considerably affected the drift of functional polymorphisms in the introduced AL population.

### Genetic differentiation between populations

As expected given the extremely short time of divergence separating the two house finch populations, the vast majority of genes showed no substantial genetic differentiation between AZ and AL. However, a few genes (*n* = 76) showed considerable allele frequency differences reflected in *F*_*ST*_ values > 3 standard deviations higher than the arithmetic mean and these could potentially constitute targets for directional selection in the disease exposed AL population (Figure 7). This set of highly differentiated genes was, however, not enriched for any particular functional category. There were five genes in which two or more non-synonymous SNPs were fixed between the house finch populations, of which 2 have unknown functions in birds, one is associated with double-strand break repair and two were associated with immune response. The latter two were a heat-shock protein and a T-cell precursor which corresponds well with the assumed strong selection for the immune response to adapt to the encounter of the novel pathogen MG [17,24,25,27,53]. It should, however, be noted that inference of differentiation from pooled RNA samples might be biased by instances of allele-specific expression and verification experiments are needed to establish candidate genes detected from differentiation scans of this type.

### Alternative splicing

Functional polymorphisms may involve primary gene and protein sequences or regulatory changes that affect the expression [54] and recently it has been recognized that, in addition, traits can be controlled via alternative splicing, spatial or temporal variation in the use of different gene transcripts [55], a process that considerably increases gene product complexity [56,57]. Recent analyses suggest that most eukaryotic genes have at least two splice variants [55,58]. Alternative splicing seems to be more common in higher eukaryotes [56,59] but results are not consistent and few taxonomic groups have been thoroughly investigated [60-62]. In order to assess the prevalence of alternative splice variation in the house finch spleen transcriptome we focused on a set of genes with a > 300 bp long uninterrupted ORF and reciprocal blast-supported 1:1 orthology to zebra finch genes (>90% identity required), resulting in a gene set of 9,167 and 9,007 annotated zebra finch genes in the AL and the AZ populations, respectively. More than half of these showed evidence of only one splice variant in both populations and the remaining genes varied between two to 23 splice isoforms. This observation is within the range of splice variation described from other vertebrates. However, it should be noted that splice variant detection using software designed for assembly may misestimate the number of isoforms [63] and additional biases might be introduced by transcript redundancy among compared datasets [60,61]. Evolutionarily novel splice variants may constitute an important source for evolution of novel functions because they might be under relatively low constraints [64]; for example, species-specific splice variants are positively correlated with non-synonymous substitution rate [65] and minor alternative exons evolve faster than obligate exons [66,67]. Our analysis revealed more than 150 unique splice variants across both populations. A conservative interpretation is that rather than reflecting divergence in inter-population splice variation, this result may reveal the challenges with reliably inferring splice variation in previously uncharacterized genomes using short-read data [63]. In order to more rigorously assess the patterns of splice variation among populations, we therefore suggest that future transcriptomes in house finches should be conducted with technologies with longer read lengths, a feature that will be increasingly feasible given the rapid developments in sequencing technology [63]. Still, it is intriguing to speculate on the evolutionary significance of alternative splicing in this system. In the first study of differential gene expression between experimentally infected and uninfected house finches [23], one of the most highly up-regulated genes as a result of infection was an alternative splicing factor, now called *SREK1* (splicing regulatory glutamine/lysine-rich protein 1, chicken ortholog: ENSGALG00000014775), whose function is to regulate alternative splicing. It is fascinating to speculate that this protein might marshal an array of functionally relevant alternatively spliced variants upon MG infection, the results of which we may be detecting in our study. In general we suspect that splice variants could be of interest for forthcoming studies on microevolutionary change in house finches and other birds.

## Conclusions

The characterization of the spleen transcriptome of the house finch will facilitate forthcoming genomic efforts in this species. By using SNPs in a large set of genes, association analyses and QTL mapping efforts can provide insight into the short-term evolutionary processes governing allele frequency changes as a consequence of putative bottlenecks, disease exposure and/or sexual selection. In addition, this resource will enhance the power of comparative genomics approaches to identify genes of importance for lineage specific adaptations and to investigate molecular evolutionary patterns across diverging lineages. In summary, this resource has paved the way to take a model species for host pathogen interactions and sexual selection into the realm of genomics.

## Methods

### Sampling, library construction and sequencing

In February 2010, three male and three female house finches were sampled from each of two populations (n = 24 chromosomes in total); one population from Green Valley, AZ (hereafter AZ), which is within the native range and historically unexposed to *Mycoplasma gallisepticum,* and one population descended from the introduced, eastern US population and previously exposed in nature to MG from Auburn, AL (hereafter AL; see [68] for a description of the MG epizootic in Auburn Alabama). Wild birds with no symptoms of MG from both populations were caught in the field using feeder traps. Immediately after capture, birds were sacrificed and the spleen was sampled and stored in RNA later (Ambion Inc., Austin, TX) at room temperature for one day and then subsequently at -80°C. We used the RNeasy Mini Kit (Qiagen, Inc., Valencia, CA) to extract total RNA from each individual spleen. However, because a single spleen did not generate enough RNA for constructing an Illumina HiSeq sequencing library we pooled individuals from each population in equimolar concentrations based on the individual RNA concentrations as measured on a Bioanalyzer (Agilent Technologies, Inc., Clara, CA). The manufacturer’s mRNA sequencing sample preparation guide (Illumina, Inc., San Diego, CA) was used to prepare two pools of total RNA for paired-end sequencing (101 bp read length). cDNA library preparation qualities were assessed with Bioanalyzer runs (Agilent Technologies, Inc., Clara, CA). To evaluate the frequency of erroneous adapter constructs we cloned (pGEM®-T Systems, Promega, Inc., Madison, WI) and sequenced 48 sample clones from each pool on a 96 capillary ABI 3730xl instrument (Life Technologies Corp., Carlsbad, CA). Seven of the 96 sequenced clones showed inaccurate adapter constructs, always in the form of one incomplete adapter. To optimize the sample concentrations and volumes for HiSeq sequencing we used a standard qPCR protocol for Illumina library preparations (KAPA library Quant Kit, Kapa Biosystems, Woburn, MA). Both pools were sequenced with Illumina HiSeq technology (Illumina, Inc., San Diego, CA) at the core facility of the FAS Center for Systems Biology at Harvard University. All sequence reads have been deposited in the sequence reads archive under accession number SRP018959 [31].

### Quality control and read filtering

We assessed overall quality and sequence read statistics using Unix shell, perl (http://www.perl.org/) and python (http://www.python.org/) scripts developed in-house in addition to the FastQC (available from http://www.bioinformatics.babraham.ac.uk/projects/fastqc/) and FastX (available from http://hannonlab.cshl.edu/fastx_toolkit/index.html) packages. Read trimming and purging of low quality bases (phred score < 25) was done using the ConDeTri program (available from http://code.google.com/p/condetri/) version 2.2 [69] (Figure 1).

### Transcript assembly

Quality-filtered reads were assembled using Trinity, a *de novo* assembler designed to efficiently and robustly reconstruct a transcriptome [70]. Trinity partitions the sequence data into individual de Bruijn graph clusters and processes them in parallel, making the assembly process relatively inexpensive computationally. In addition to generating the longest transcript sequence, possible splice isoforms were automatically reconstructed for each gene using default settings in Trinity (Figure 1).

### Filters of assembled transcripts

Because the primary aim of the study was to assess gene expression differences and genetic differentiation between house finch populations, we focused primarily on protein-coding genes and a series of filters were therefore applied to remove potentially confounding sequences like non-coding RNA species, expressed pseudogenes and transcripts of unknown function [71]. First, transcripts with short length were excluded. To find a reasonable size threshold for including a transcript in the data set, the mean size (*μ =* 956 bp) and standard deviation (s.d. = 494 bp) of all known cDNA sequences in zebra finch Ensembl release [69,72] was calculated. We used *μ* - 1.s.d. (462 bp) as the cutoff and applied the threshold to assembled house finch transcripts. Second, we searched for intact open reading frames (ORFs) in these filtered house finch transcripts, using the Trinity tool suite and only transcripts with predicted ORFs longer than 300 bp were kept; we define this set as the unfiltered set. Finally, we mapped the unfiltered set to all known zebra finch cDNAs Ensembl release [69,72] by BLAT [33], and positive hits were denoted as high-confidence house finch coding transcripts, and retained for primary analyses, if the alignment covered ≥ 60% of the total zebra finch transcript length with ≥ 80% identity. We define this set as the filtered set. The corresponding transcripts of the house finch filtered set in zebra finch we designate as zebra finch orthologs. Our filtering steps inevitably biased the dataset towards conserved genes (high-degree of similarity between species) of intermediate length since rapidly evolving genes (>20% divergence between species) and short (<300 bp coding sequence) or long (lower chance of spanning > 60% of the entire gene length) genes are more likely filtered out using these criteria (Figure 1). We also identified orthologs of the filtered gene set in chicken, *Anolis* lizard and in humans using similar protocols, but requiring at least 60%, 50% and 30% sequence identities in chickens, *Anolis* and humans, respectively, to account for their increasing evolutionary divergence from house finches.

### Analysis of differential gene expression between populations

We mapped all trimmed reads to the *de novo* assembled house finch transcripts and calculated both raw read counts and transcripts per million reads (TPM) for each transcript using the RSEM software [36]. Subsequently, the DESeq package [37] was used to identify differentially expressed genes (DEG) between the two house finch populations (AZ and AL). Transcripts at extremely low expression level (<1 read count per million reads (CPM) identified using edgeR: a bioconductor package for differential expression analysis of digital gene expression data) were excluded. We used CPM here instead of TPM to filter minimally expressed transcripts because with TPM some short transcripts with questionably low expression may not be detected due to the normalization based on transcript length. DEGs were defined as transcripts with a *p*-value < 0.05 after applying the Benjamini-Hochberg adjustment of the significance level [73] (Figure 1).

### Identifying single nucleotide polymorphisms (SNPs) and estimating nucleotide diversity

To identify single nucleotide polymorphisms (SNPs) within house finch transcripts, we mapped trimmed reads to the filtered transcripts using Bowtie 2 [74], using the default parameter set. Only alignments with a quality score ≥ 30 were retained. SAMtools [75] was used to call SNPs in the alignments using a coverage threshold of at least five reads overlapping a given SNP position and a minor allele frequency (MAF) for the SNP ≥ 0.05. In cases where two SNPs occurred within five base pairs of each other, both SNPs were discarded so as to decrease the number of erroneous polymorphic sites called due to misalignments (Figure 1). We estimated the nucleotide diversity using the method of Watterson [39] (Equation 1)

(1) $$\Theta_{W}=\frac{S_{n}}{L\sum_{j}^{n-1}\frac{1}{j}}$$

where *S*_*n*_ is the number of segregating sites, *n* is the number of sequences, and *L* is the length of a given sequence.

### Estimating rates of nonsynonymous substitution (p_N_/p_S_)

SNPs were classified as non-coding, synonymous or non-synonymous, according to their positions in the predicted ORFs, and synonymous and non-synonymous sites were identified for each ORF using the method of Nei and Gojobori [76] on the filtered data set. These sites were used to estimate the ratio of non-synonymous (*p*_*N*_) to synonymous polymorphisms (*p*_*S*_) for each transcript and each population, by using perl scripts developed in house and calculating the number of non-synonymous SNPs divided by the number of non-synonymous sites and the number of synonymous SNPs divided by the number of synonymous sites (Figure 1).

### Estimating genetic differentiation (F_ST_)

To characterize population differentiation, we also calculated *F*_*ST*_[77] between the AL and AZ populations for each transcript, using a simple estimate of the proportions of the nucleotide diversity present within and between populations (Equation 2), where *π*_*between*_ denotes the average number of pair-wise differences for a specific transcript between populations and *π*_*within*_ denotes the average number of pair-wise differences for the same transcript within populations.

(2) $$F_{\mathrm{ST}}=\frac{\pi_{\mathrm{between}}‒\pi_{\mathrm{within}}}{\pi_{\mathrm{between}}}$$

To enhance the reliability of transcript-specific estimates we excluded transcripts with less than three SNPs.

### Gene ontology analysis

Since there is no annotation for house finch genes currently available, we used the gene ontology [78] annotations of zebra finch orthologs retrieved using Ensembl’s Biomart release 69 [79]. Tests for functional enrichment in sets of transcripts either differentially expressed or exhibiting high genetic differentiation between house finch populations were then conducted using TopGO [80], a software package that compares the difference in occurrences in a given functional category between foreground and background sets of transcripts and assesses significance using Fisher’s exact test (Figure 1). Correction for multiple tests was again performed using the Benjamini-Hochberg approach [73].

## Competing interests

The authors declare that they have no competing interests.

## Authors’ contributions

NB and SVE conceived of the study and planned the analyses. NB collected samples in the field, performed the molecular work and carried out parts of the computational analyses. QZ carried out the bioinformatic analyses and performed the statistical analyses. NB and QZ drafted the manuscript. GEH participated in the coordination of field work. All authors read, commented on and approved the final manuscript.

## Supplementary Material

Additional file 1 Supplementary information. Click here for file
