# Supplementary material for: A house finch (Haemorhous mexicanus) spleen transcriptome reveals intra- and interspecific patterns of gene expression, alternative splicing and genetic diversity in passerines
Source: BMC Genomics. 2014 Apr 24;15:305. doi: 10.1186/1471-2164-15-305 (PMC4235107; doi:10.1186/1471-2164-15-305)
Supplement: Additional file 1 — Supplementary information. [file 1471-2164-15-305-S1.pdf]

Supplementary Information

Supplementary Table 1.

Assembly/filtering steps	Trinity assembly	Size filter (>462 bp)[1]	Uninterrupted ORF >300bp	Zebra finch ortholog[2]
# Contigs / genes	222,678	82,384	47,542	9,646
Contigs / genes %	100	37	21	4

[1] Average cDNA length in the zebra finch – 1 standard deviation (956-462).

[2] Criteria: > 300 bp length, >80% sequence similarity between house finch and zebra finch and > 60% of the total gene length of the zebra finch gene.

Supplementary Table 2.

Summary of the number of reads before and after quality filtering for each of the two house finch populations (AL and AZ) sequenced.

Dataset	Total # reads	Total # bp before filtering	# filtered reads with paired-end information	# filtered reads without paired-end information	Total # filtered reads	Total # bp after filtering
AL HiSeq	251,064,928[1]	25,357,557,728	145,022,154[3]	36,398,704	181,420,858	15,857,600,030*
AZ HiSeq	250,854,478[2]	25,336,302,278	147,075,694[4]	35,950,613	183,026,307	16,038,772,338**

[1]125,532,464*2 reads
[2]125,427,239*2 reads
[3]72,511,077*2 reads
[4]73,537,847*2 reads
* 6_1b_trim1 = 6,745,595,114 bp, 6_1b_trim2 = 6,147,325,369 bp, 6_1b_trim_unpaired = 2,964,679,547 bp
**7_1b_trim1 = 6,842,605,767 bp, 7_1b_trim2 = 6,271,042,361, 7_1b_trim_unpaired = 2,925,124,210 bp

Supplementary Table 3.

Gene ontology terms for genes uniquely expressed in house finch and zebra finch when comparing expression profiles of the spleen. MF = molecular function, CC = cellular component. GO.ID is the identification number for the gene ontology term and given is also the annotated, significant and expected number of genes for each term and the uncorrected (Classic p) and corrected (Corrected p) p-values.

GO.ID	Term	Annotated	Significant	Expected	Classic p	Corrected p
**house finch-specific**						
GO:0003824 (MF)	catalytic activity	3468	713	633	$4.5*10^{-6}$	0.006
GO:0005515 (MF)	protein binding	5002	984	913	$4.8*10^{-5}$	0.032
GO:0042802 (MF)	identical protein binding	320	86	58	$6.9*10^{-5}$	0.032
GO:0050662 (MF)	coenzyme binding	142	45	26	$7.1*10^{-5}$	0.032
GO:0051287 (MF)	NAD binding	30	15	5	$7.7*10^{-5}$	0.032
GO:0005737 (CC)	cytoplasm	3250	676	597	$4.1*10^{-7}$	0.000
GO:0044444 (CC)	cytoplasmic part	2261	485	415	$2.5*10^{-6}$	0.001
**zebra finch-specific**						
GO:0003735 (MF)	structural constituent of ribosome	126	40	22	$9.4*10^{-5}$	0.041
GO:0004129 (MF)	cytochrome-c oxidase activity	11	8	2	$9.8*10^{-5}$	0.041
GO:0008009 (MF)	chemokine activity	11	8	2	$9.8*10^{-5}$	0.041
GO:0015002 (MF)	heme-copper terminal oxidase activity	11	8	2	$9.8*10^{-5}$	0.041
GO:0016675 (MF)	oxidoreductase activity	11	8	2	$9.8*10^{-5}$	0.041
GO:0016676 (MF)	oxidoreductase activity	11	8	2	$9.8*10^{-5}$	0.041

Supplementary Table 4.

Gene identification details for genes differentially expressed between the two house finch populations when comparing expression profiles of the spleen between populations. Given is the transcript and gene ID numbers for zebra finch orthologs, a description of the function, the observed fold change (FC; Log 2 scaled; AZ is the reference population) also and the uncorrected (Classic p) and corrected (Corrected p) p-values. Inf = infinite (gene not expressed in one population).

ZF ortholog transcript	Ensembl Gene ID	Description	FC	Classic p	Corrected p
ENSTGUT00000000012	ENSTGUG00000000014	Uncharacterized protein	Inf	$3.8*10^{-7}$	$5.0*10^{-5}$
ENSTGUT00000000296	ENSTGUG00000000288	ubiquitin domain containing 2	Inf	$1.4*10^{-4}$	$9.4*10^{-3}$
ENSTGUT00000000450	ENSTGUG00000000437	nuclear receptor binding SET domain	Inf	$1.1*10^{-4}$	$7.5*10^{-3}$
ENSTGUT00000001085	ENSTGUG00000001047	Uncharacterized protein	Inf	$4.0*10^{-4}$	$2.4*10^{-2}$
ENSTGUT00000001295	ENSTGUG00000001241	NOP2/Sun domain family, member 6	Inf	$1.5*10^{-4}$	$1.0*10^{-2}$
ENSTGUT00000001337	ENSTGUG00000001279	KIAA0368	Inf	$9.3*10^{-12}$	$2.7*10^{-9}$
ENSTGUT00000001405	ENSTGUG00000001350	Uncharacterized protein	Inf	$7.0*10^{-5}$	$5.2*10^{-3}$
ENSTGUT00000001496	ENSTGUG00000001421	kinase complex-associated protein [	Inf	$3.8*10^{-12}$	$1.3*10^{-9}$
ENSTGUT00000002339	ENSTGUG00000002251	leucine rich repeat containing 3C	Inf	$6.7*10^{-15}$	$2.9*10^{-12}$
ENSTGUT00000002427	ENSTGUG00000002339	transmembrane protein 141	Inf	$6.1*10^{-5}$	$4.7*10^{-3}$
ENSTGUT00000002494	ENSTGUG00000002405	KIAA2013	Inf	$5.3*10^{-25}$	$7.9*10^{-22}$
ENSTGUT00000002513	ENSTGUG00000002421	Molybdopterin synthase	Inf	$7.6*10^{-7}$	$9.3*10^{-5}$
ENSTGUT00000002546	ENSTGUG00000002433	Uncharacterized protein	Inf	$4.7*10^{-18}$	$3.0*10^{-15}$
ENSTGUT00000003786	ENSTGUG00000003626	eukaryotic translation initiation factor	Inf	$7.6*10^{-10}$	$1.8*10^{-7}$
ENSTGUT00000003886	ENSTGUG00000003728	BARX homeobox 1	Inf	$5.0*10^{-4}$	$2.8*10^{-2}$
ENSTGUT00000004383	ENSTGUG00000004168	dihydropyrimidinase-like 2	Inf	$1.9*10^{-15}$	$9.2*10^{-13}$
ENSTGUT00000005083	ENSTGUG00000004896	malignant fibrous histiocytoma	Inf	$1.4*10^{-5}$	$1.4*10^{-3}$
ENSTGUT00000005239	ENSTGUG00000005019	phosphoadenosine phosphosulfate synthase	Inf	$1.1*10^{-17}$	$6.5*10^{-15}$
ENSTGUT00000005279	ENSTGUG00000005066	ubiquitin-like domain member 1	Inf	$2.6*10^{-28}$	$4.6*10^{-25}$
ENSTGUT00000006163	ENSTGUG00000005932	Uncharacterized protein	Inf	$5.0*10^{-4}$	$2.8*10^{-2}$
ENSTGUT00000006667	ENSTGUG00000006371	Uncharacterized protein	Inf	$3.9*10^{-13}$	$1.5*10^{-10}$

ENSTGUT00000006970	ENSTGUG00000006705	immunoglobulin mu binding protein 2	Inf	$9.3*10^{-11}$	$2.4*10^{-8}$
ENSTGUT00000007101	ENSTGUG00000006831	claudin 18	Inf	$1.0*10^{-20}$	$9.0*10^{-18}$
ENSTGUT00000007163	ENSTGUG00000006895	blocked early in transport 1 homolog	Inf	$9.6*10^{-8}$	$1.5*10^{-5}$
ENSTGUT00000007323	ENSTGUG00000007041	Uncharacterized protein	Inf	$3.9*10^{-8}$	$6.4*10^{-6}$
ENSTGUT00000007384	ENSTGUG00000007093	coiled-coil domain containing 66	Inf	$4.6*10^{-4}$	$2.7*10^{-2}$
ENSTGUT00000008451	ENSTGUG00000008112	tweety homolog 3 (Drosophila)	Inf	$1.3*10^{-9}$	$2.9*10^{-7}$
ENSTGUT00000008764	ENSTGUG00000008397	Uncharacterized protein	Inf	$1.1*10^{-7}$	$1.7*10^{-5}$
ENSTGUT00000009430	ENSTGUG00000009058	Uncharacterized protein	Inf	$2.4*10^{-4}$	$1.5*10^{-2}$
ENSTGUT00000009893	ENSTGUG00000009497	Uncharacterized protein	Inf	$1.3*10^{-7}$	$1.9*10^{-5}$
ENSTGUT00000010476	ENSTGUG00000010043	adenosine A2a receptor	Inf	$7.4*10^{-13}$	$2.7*10^{-10}$
ENSTGUT00000010872	ENSTGUG00000010404	HEAT repeat containing 1	Inf	$1.2*10^{-6}$	$1.4*10^{-4}$
ENSTGUT00000010902	ENSTGUG00000010462	spermatogenesis associated, serine-rich	Inf	$7.1*10^{-6}$	$7.2*10^{-4}$
ENSTGUT00000011050	ENSTGUG00000010605	dual specificity phosphatase 5	Inf	$3.7*10^{-5}$	$3.0*10^{-3}$
ENSTGUT00000011568	ENSTGUG00000011096	sorting nexin	Inf	$3.1*10^{-9}$	$6.4*10^{-7}$
ENSTGUT00000011580	ENSTGUG00000011114	inositol monophosphatase domain containing	Inf	$5.8*10^{-12}$	$1.7*10^{-9}$
ENSTGUT00000011769	ENSTGUG00000011296	nucleoporin 37kDa	Inf	$6.2*10^{-4}$	$3.4*10^{-2}$
ENSTGUT00000012198	ENSTGUG00000011708	ubiquitin specific peptidase like 1	Inf	$2.8*10^{-8}$	$5.0*10^{-6}$
ENSTGUT00000012599	ENSTGUG00000012089	signal transducing adaptor molecule	Inf	$5.5*10^{-22}$	$5.5*10^{-19}$
ENSTGUT00000013124	ENSTGUG00000012592	pleckstrin homology domain	Inf	$3.0*10^{-7}$	$4.1*10^{-5}$
ENSTGUT00000016728	ENSTGUG00000016092	Uncharacterized protein	Inf	$5.8*10^{-12}$	$1.7*10^{-9}$
ENSTGUT00000017460	ENSTGUG00000016801	uncharacterized protein	Inf	$8.3*10^{-20}$	$6.2*10^{-17}$
ENSTGUT00000017933	ENSTGUG00000017252	flavin containing monooxygenase 5	Inf	$1.4*10^{-5}$	$1.4*10^{-3}$
ENSTGUT00000017935	ENSTGUG00000017256	phosphatidic acid phosphatase	Inf	$3.3*10^{-9}$	$6.6*10^{-7}$
ENSTGUT00000017942	ENSTGUG00000017264	Fas ligand	Inf	$4.1*10^{-5}$	$3.3*10^{-3}$
ENSTGUT00000019270	ENSTGUG00000018128	leucine rich repeat containing 23	Inf	$8.9*10^{-8}$	$1.4*10^{-5}$
ENSTGUT00000004150	ENSTGUG00000003990	gastrokine 2	12.2	$1.0*10^{-55}$	$4.7*10^{-52}$
ENSTGUT00000009695	ENSTGUG00000009299	Uncharacterized protein	11.4	$2.7*10^{-30}$	$6.0*10^{-27}$
ENSTGUT00000000592	ENSTGUG00000000572	serine peptidase inhibitor	10.8	$1.7*10^{-57}$	$1.5*10^{-53}$
ENSTGUT00000007105	ENSTGUG00000006846	acetylglucosaminyltransferase	8.7	$1.5*10^{-8}$	$2.8*10^{-6}$
ENSTGUT00000009926	ENSTGUG00000009524	diacylglycerol kinase, epsilon 64kDa	8.3	$4.2*10^{-7}$	$5.5*10^{-5}$

ENSTGUT00000001865	ENSTGUG00000001793	Motilin	7.7	$1.9*10^{-8}$	$3.5*10^{-6}$
ENSTGUT00000018192	ENSTGUG00000017507	cathepsin E	7.4	$1.3*10^{-31}$	$3.8*10^{-28}$
ENSTGUT00000006046	ENSTGUG00000005823	galactose-3-O-sulfotransferase 4	6.0	$4.6*10^{-4}$	$2.7*10^{-2}$
ENSTGUT00000010432	ENSTGUG00000009979	Janus kinase 1	5.5	$4.9*10^{-24}$	$6.1*10^{-21}$
ENSTGUT00000018139	ENSTGUG00000017454	chitinase, acidic	5.4	$5.4*10^{-24}$	$6.1*10^{-21}$
ENSTGUT00000002705	ENSTGUG00000002599	Uncharacterized protein	5.3	$8.1*10^{-4}$	$4.2*10^{-2}$
ENSTGUT00000011183	ENSTGUG00000010726	Smoothelin	5.3	$7.1*10^{-16}$	$3.8*10^{-13}$
ENSTGUT00000004895	ENSTGUG00000004696	chloride intracellular channel 6	5.2	$1.6*10^{-6}$	$1.8*10^{-4}$
ENSTGUT00000009942	ENSTGUG00000009537	Uncharacterized protein	5.0	$1.5*10^{-17}$	$8.5*10^{-15}$
ENSTGUT00000004023	ENSTGUG00000003867	SIX homeobox 2	4.8	$8.4*10^{-7}$	$1.0*10^{-4}$
ENSTGUT00000007449	ENSTGUG00000007145	microtubule associated tumor suppressor 1	4.5	$1.1*10^{-7}$	$1.7*10^{-5}$
ENSTGUT00000011038	ENSTGUG00000010566	Uncharacterized protein	4.4	$4.2*10^{-11}$	$1.1*10^{-8}$
ENSTGUT00000001728	ENSTGUG00000001661	immunoglobulin J	4.3	$5.2*10^{-15}$	$2.3*10^{-12}$
ENSTGUT00000007386	ENSTGUG00000007067	Uncharacterized protein	4.3	$4.1*10^{-18}$	$2.8*10^{-15}$
ENSTGUT00000004230	ENSTGUG00000004066	V-set and immunoglobulin domain	4.2	$2.8*10^{-5}$	$2.4*10^{-3}$
ENSTGUT00000006705	ENSTGUG00000006454	myosin, light chain 2	4.2	$8.5*10^{-4}$	$4.3*10^{-2}$
ENSTGUT00000005362	ENSTGUG00000005137	Uncharacterized protein	4.1	$4.3*10^{-13}$	$1.6*10^{-10}$
ENSTGUT00000008727	ENSTGUG00000008359	Uncharacterized protein	4.1	$2.9*10^{-7}$	$4.1*10^{-5}$
ENSTGUT00000008462	ENSTGUG00000008121	LFNG acetylglucosaminyltransferase	4.0	$8.4*10^{-14}$	$3.4*10^{-11}$
ENSTGUT00000000688	ENSTGUG00000000665	Fc fragment of IgG binding protein	4.0	$1.6*10^{-5}$	$1.5*10^{-3}$
ENSTGUT00000008686	ENSTGUG00000008341	Uncharacterized protein	4.0	$2.3*10^{-8}$	$4.1*10^{-6}$
ENSTGUT00000007551	ENSTGUG00000007197	Uncharacterized protein	4.0	$8.4*10^{-16}$	$4.2*10^{-13}$
ENSTGUT00000003660	ENSTGUG00000003504	solute carrier family 25	3.5	$2.5*10^{-12}$	$8.7*10^{-10}$
ENSTGUT00000013322	ENSTGUG00000012795	BAG family molecular chaperone	3.3	$1.5*10^{-4}$	$1.0*10^{-2}$
ENSTGUT00000008009	ENSTGUG00000007683	stearoyl-CoA desaturase	3.3	$4.1*10^{-12}$	$1.3*10^{-9}$
ENSTGUT00000005986	ENSTGUG00000005766	tubulin polymerization-promoting protein	3.2	$8.2*10^{-4}$	$4.2*10^{-2}$
ENSTGUT00000005750	ENSTGUG00000005378	myosin, heavy chain 11	3.2	$1.9*10^{-7}$	$2.7*10^{-5}$
ENSTGUT00000007970	ENSTGUG00000007603	protein phosphatase 1	3.2	$5.9*10^{-7}$	$7.6*10^{-5}$
ENSTGUT00000002938	ENSTGUG00000002828	Uncharacterized protein	3.2	$1.9*10^{-11}$	$5.4*10^{-9}$
ENSTGUT00000014904	ENSTGUG00000014295	alanyl (membrane) aminopeptidase	3.1	$7.4*10^{-10}$	$1.8*10^{-7}$

ENSTGUT00000007385	ENSTGUG00000007082	serine hydroxymethyltransferase 1	3.0	$1.0*10^{-10}$	$2.5*10^{-8}$
ENSTGUT00000016883	ENSTGUG00000016242	Uncharacterized protein	3.0	$8.1*10^{-11}$	$2.1*10^{-8}$
ENSTGUT00000007957	ENSTGUG00000007617	ATP-binding cassette, sub-family B	3.0	$3.5*10^{-9}$	$6.8*10^{-7}$
ENSTGUT00000012706	ENSTGUG00000012201	regulator of chromosome condensation	2.8	$3.6*10^{-5}$	$3.0*10^{-3}$
ENSTGUT00000008127	ENSTGUG00000007772	Uncharacterized protein	2.8	$1.9*10^{-9}$	$4.0*10^{-7}$
ENSTGUT00000011545	ENSTGUG00000011059	Uncharacterized protein	2.8	$3.7*10^{-8}$	$6.3*10^{-6}$
ENSTGUT00000006415	ENSTGUG00000006167	S-adenosylmethionine synthase	2.7	$8.1*10^{-9}$	$1.5*10^{-6}$
ENSTGUT00000013395	ENSTGUG00000012861	creatine kinase, brain	2.6	$3.5*10^{-8}$	$6.0*10^{-6}$
ENSTGUT00000001079	ENSTGUG00000001028	Supervillin	2.6	$5.1*10^{-4}$	$2.9*10^{-2}$
ENSTGUT00000003535	ENSTGUG00000003373	phosphoserine aminotransferase 1	2.6	$3.8*10^{-8}$	$6.3*10^{-6}$
ENSTGUT00000002443	ENSTGUG00000002351	tensin 4	2.5	$3.4*10^{-4}$	$2.1*10^{-2}$
ENSTGUT00000013630	ENSTGUG00000013085	lipin 1	2.5	$2.8*10^{-6}$	$3.0*10^{-4}$
ENSTGUT00000006204	ENSTGUG00000005956	CAP, adenylate cyclase	2.5	$4.9*10^{-5}$	$3.9*10^{-3}$
ENSTGUT00000012699	ENSTGUG00000012193	uridine phosphorylase 2	2.4	$1.9*10^{-7}$	$2.7*10^{-5}$
ENSTGUT00000003551	ENSTGUG00000003390	5'-nucleotidase domain containing 4	2.4	$1.4*10^{-7}$	$2.1*10^{-5}$
ENSTGUT00000010515	ENSTGUG00000010074	Uncharacterized protein	2.4	$2.2*10^{-4}$	$1.5*10^{-2}$
ENSTGUT00000000183	ENSTGUG00000000175	Transgelin	2.3	$3.5*10^{-7}$	$4.8*10^{-5}$
ENSTGUT00000003267	ENSTGUG00000003144	hematopoietic prostaglandin D	2.3	$5.6*10^{-4}$	$3.1*10^{-2}$
ENSTGUT00000008883	ENSTGUG00000008503	Histidine ammonia-lyase	2.2	$1.7*10^{-4}$	$1.1*10^{-2}$
ENSTGUT00000001677	ENSTGUG00000001614	Uncharacterized protein	2.2	$2.6*10^{-5}$	$2.3*10^{-3}$
ENSTGUT00000017922	ENSTGUG00000017244	Uncharacterized protein	2.2	$7.4*10^{-7}$	$9.2*10^{-5}$
ENSTGUT00000004035	ENSTGUG00000003719	fatty acid synthase	2.2	$1.0*10^{-6}$	$1.2*10^{-4}$
ENSTGUT00000008570	ENSTGUG00000008074	acetyl-CoA carboxylase alpha	2.2	$1.4*10^{-6}$	$1.6*10^{-4}$
ENSTGUT00000005126	ENSTGUG00000004885	glycine dehydrogenase	2.2	$1.7*10^{-6}$	$2.0*10^{-4}$
ENSTGUT00000004251	ENSTGUG00000004052	Plectin	2.2	$3.7*10^{-6}$	$3.9*10^{-4}$
ENSTGUT00000004703	ENSTGUG00000004509	beta-carotene 15,15'-monooxygenase	2.2	$4.5*10^{-5}$	$3.6*10^{-3}$
ENSTGUT00000002311	ENSTGUG00000002207	complement component 6	2.2	$3.6*10^{-5}$	$3.0*10^{-3}$
ENSTGUT00000009618	ENSTGUG00000009234	matrix Gla protein	2.2	$2.4*10^{-6}$	$2.7*10^{-4}$
ENSTGUT00000018845	ENSTGUG00000018178	Uncharacterized protein	2.1	$2.1*10^{-5}$	$1.9*10^{-3}$
ENSTGUT00000006350	ENSTGUG00000006111	4-hydroxyphenylpyruvate dioxygenase	2.1	$2.6*10^{-6}$	$2.8*10^{-4}$

ENSTGUT00000008218	ENSTGUG00000007790	Uncharacterized protein	2.1	$4.8*10^{-6}$	$5.0*10^{-4}$
ENSTGUT00000000451	ENSTGUG00000000430	Uncharacterized protein	2.1	$1.1*10^{-4}$	$7.4*10^{-3}$
ENSTGUT00000002773	ENSTGUG00000002669	Uncharacterized protein	2.1	$6.0*10^{-6}$	$6.2*10^{-4}$
ENSTGUT00000013600	ENSTGUG00000013059	tsukushi small leucine rich proteoglycan	2.0	$1.6*10^{-4}$	$1.1*10^{-2}$
ENSTGUT00000001125	ENSTGUG00000001083	selenoprotein N, 1 precursor	2.0	$2.6*10^{-4}$	$1.6*10^{-2}$
ENSTGUT00000005307	ENSTGUG00000005087	cholesteryl ester transfer protein, plasma	2.0	$2.2*10^{-5}$	$2.0*10^{-3}$
ENSTGUT00000017147	ENSTGUG00000016499	myosin, light chain 9, regulatory	2.0	$1.3*10^{-5}$	$1.3*10^{-3}$
ENSTGUT00000013348	ENSTGUG00000012819	ankyrin repeat domain 9	2.0	$5.8*10^{-5}$	$4.5*10^{-3}$
ENSTGUT00000004245	ENSTGUG00000004067	Uncharacterized protein	1.9	$3.7*10^{-4}$	$2.3*10^{-2}$
ENSTGUT00000009632	ENSTGUG00000009192	acyl-CoA oxidase 2, branched chain	1.9	$5.0*10^{-4}$	$2.8*10^{-2}$
ENSTGUT00000008464	ENSTGUG00000008105	transmembrane protein 63B	1.9	$1.3*10^{-4}$	$8.7*10^{-3}$
ENSTGUT00000014074	ENSTGUG00000013515	prostaglandin F2 receptor	1.9	$7.8*10^{-4}$	$4.1*10^{-2}$
ENSTGUT00000008599	ENSTGUG00000008257	phosphoribosyl pyrophosphate	1.9	$2.2*10^{-5}$	$2.0*10^{-3}$
ENSTGUT00000006311	ENSTGUG00000006062	Desmin	1.9	$2.0*10^{-5}$	$1.9*10^{-3}$
ENSTGUT00000000498	ENSTGUG00000000472	phosphoribosylglycinamide synthetase	1.9	$2.3*10^{-5}$	$2.1*10^{-3}$
ENSTGUT00000008203	ENSTGUG00000007885	Uncharacterized protein	1.9	$2.2*10^{-5}$	$2.0*10^{-3}$
ENSTGUT00000001946	ENSTGUG00000001872	tropomyosin 4	1.9	$2.8*10^{-5}$	$2.4*10^{-3}$
ENSTGUT00000002677	ENSTGUG00000002578	anterior gradient 2 homolog	1.9	$9.2*10^{-4}$	$4.6*10^{-2}$
ENSTGUT00000010707	ENSTGUG00000010247	Uncharacterized protein	1.8	$3.8*10^{-4}$	$2.3*10^{-2}$
ENSTGUT00000005296	ENSTGUG00000005086	Uncharacterized protein	1.8	$3.9*10^{-5}$	$3.2*10^{-3}$
ENSTGUT00000003093	ENSTGUG00000002942	IMP cyclohydrolase	1.8	$5.4*10^{-5}$	$4.2*10^{-3}$
ENSTGUT00000009863	ENSTGUG00000009472	CCAAT/enhancer binding protein	1.8	$6.7*10^{-5}$	$5.0*10^{-3}$
ENSTGUT00000010552	ENSTGUG00000010111	adenylosuccinate lyase	1.8	$7.6*10^{-5}$	$5.6*10^{-3}$
ENSTGUT00000011745	ENSTGUG00000011260	lanosterol synthase	1.8	$2.2*10^{-4}$	$1.4*10^{-2}$
ENSTGUT00000005411	ENSTGUG00000005210	glycine N-methyltransferase	1.8	$8.7*10^{-5}$	$6.4*10^{-3}$
ENSTGUT00000011796	ENSTGUG00000011323	iodotyrosine deiodinase	1.8	$8.2*10^{-4}$	$4.2*10^{-2}$
ENSTGUT00000007701	ENSTGUG00000007393	Uncharacterized protein	1.7	$9.1*10^{-5}$	$6.6*10^{-3}$
ENSTGUT00000018026	ENSTGUG00000017345	glutamate-ammonia ligase	1.7	$8.8*10^{-5}$	$6.4*10^{-3}$
ENSTGUT00000008728	ENSTGUG00000008374	tyrosine aminotransferase	1.7	$1.0*10^{-4}$	$7.1*10^{-3}$
ENSTGUT00000011370	ENSTGUG00000010913	AIG2-like domain 1	1.7	$6.8*10^{-4}$	$3.7*10^{-2}$

ENSTGUT00000005609	ENSTGUG00000005357	Uncharacterized protein	1.7	$7.5*10^{-4}$	$4.0*10^{-2}$
ENSTGUT00000015297	ENSTGUG00000014696	transmembrane protein 98	1.7	$3.2*10^{-4}$	$2.0*10^{-2}$
ENSTGUT00000008512	ENSTGUG00000008165	tetraspanin 1	1.7	$8.3*10^{-4}$	$4.2*10^{-2}$
ENSTGUT00000007725	ENSTGUG00000007413	Amidophosphoribosyltransferase	1.7	$2.3*10^{-4}$	$1.5*10^{-2}$
ENSTGUT00000003586	ENSTGUG00000003422	formiminotransferase cyclodeaminase	1.6	$3.6*10^{-4}$	$2.2*10^{-2}$
ENSTGUT00000008843	ENSTGUG00000008491	amidohydrolase domain containing 1	1.6	$4.8*10^{-4}$	$2.8*10^{-2}$
ENSTGUT00000018154	ENSTGUG00000017469	ethanolamine kinase 2	1.6	$3.4*10^{-4}$	$2.1*10^{-2}$
ENSTGUT00000017364	ENSTGUG00000016675	Uncharacterized protein	1.6	$9.7*10^{-4}$	$4.8*10^{-2}$
ENSTGUT00000010314	ENSTGUG00000009884	phosphomannomutase 1	1.6	$6.2*10^{-4}$	$3.4*10^{-2}$
ENSTGUT00000006768	ENSTGUG00000006517	solute carrier family 38	1.6	$4.6*10^{-4}$	$2.7*10^{-2}$
ENSTGUT00000006745	ENSTGUG00000006479	dihydroxyacetone kinase 2 homolog	1.6	$4.1*10^{-4}$	$2.4*10^{-2}$
ENSTGUT00000006925	ENSTGUG00000006658	adenosine kinase	1.5	$8.3*10^{-4}$	$4.2*10^{-2}$
ENSTGUT00000013061	ENSTGUG00000012540	malic enzyme 1	1.5	$7.6*10^{-4}$	$4.0*10^{-2}$
ENSTGUT00000010080	ENSTGUG00000009626	Uncharacterized protein	1.5	$7.0*10^{-4}$	$3.8*10^{-2}$
ENSTGUT00000009203	ENSTGUG00000008832	glutamic pyruvate transaminase	1.5	$8.0*10^{-4}$	$4.2*10^{-2}$
ENSTGUT00000003947	ENSTGUG00000003783	Uncharacterized protein	1.5	$8.6*10^{-4}$	$4.3*10^{-2}$
ENSTGUT00000003733	ENSTGUG00000003532	Uncharacterized protein	-1.5	$9.5*10^{-4}$	$4.7*10^{-2}$
ENSTGUT00000011601	ENSTGUG00000011092	Uncharacterized protein	-1.6	$5.0*10^{-4}$	$2.8*10^{-2}$
ENSTGUT00000000545	ENSTGUG00000000528	DnaJ (Hsp40) homolog, subfamily C	-1.7	$5.5*10^{-4}$	$3.0*10^{-2}$
ENSTGUT00000011281	ENSTGUG00000010815	Uncharacterized protein	-1.8	$6.7*10^{-5}$	$5.0*10^{-3}$
ENSTGUT00000007086	ENSTGUG00000006791	nuclear prelamin A recognition factor	-2.1	$7.3*10^{-4}$	$3.9*10^{-2}$
ENSTGUT00000001616	ENSTGUG00000001547	guanine nucleotide binding protein	-2.2	$2.3*10^{-5}$	$2.1*10^{-3}$
ENSTGUT00000010596	ENSTGUG00000010102	Uncharacterized protein	-2.2	$2.9*10^{-4}$	$1.8*10^{-2}$
ENSTGUT00000013830	ENSTGUG00000013281	translocation associated membrane protein	-2.2	$1.6*10^{-5}$	$1.5*10^{-3}$
ENSTGUT00000016331	ENSTGUG00000015653	myosin IA	-2.3	$3.9*10^{-4}$	$2.3*10^{-2}$
ENSTGUT00000003600	ENSTGUG00000003400	phospholipase A2, group IVA	-2.5	$1.1*10^{-4}$	$7.5*10^{-3}$
ENSTGUT00000010299	ENSTGUG00000009877	sarcoma viral oncogene variant 2	-2.5	$7.3*10^{-6}$	$7.4*10^{-4}$
ENSTGUT00000006542	ENSTGUG00000006303	small nuclear RNA activating complex	-2.8	$5.5*10^{-5}$	$4.3*10^{-3}$
ENSTGUT00000001989	ENSTGUG00000001899	Uncharacterized protein	-3.4	$4.1*10^{-4}$	$2.4*10^{-2}$
ENSTGUT00000013030	ENSTGUG00000012508	putative ATPase H+ transport	-3.5	$1.8*10^{-9}$	$3.8*10^{-7}$

ENSTGUT00000001628	ENSTGUG00000001563	Uncharacterized protein	-3.6	$1.2*10^{-9}$	$2.7*10^{-7}$
ENSTGUT00000012368	ENSTGUG00000011871	cadherin 17, LI cadherin	-3.6	$6.9*10^{-7}$	$8.7*10^{-5}$
ENSTGUT00000014088	ENSTGUG00000013528	Uncharacterized protein	-3.8	$3.1*10^{-6}$	$3.3*10^{-4}$
ENSTGUT00000019201	ENSTGUG00000018460	matrix metallopeptidase 7	-4.0	$1.4*10^{-6}$	$1.6*10^{-4}$
ENSTGUT00000011164	ENSTGUG00000010720	Uncharacterized protein	-4.1	$5.5*10^{-7}$	$7.1*10^{-5}$
ENSTGUT00000002715	ENSTGUG00000002612	Uncharacterized protein	-4.3	$1.8*10^{-9}$	$3.8*10^{-7}$
ENSTGUT00000001504	ENSTGUG00000001447	rearranged L-myc fusion	-5.0	$2.7*10^{-5}$	$2.3*10^{-3}$
ENSTGUT00000006083	ENSTGUG00000005828	mitogen-activated protein kinase kinase 4	-5.1	$1.1*10^{-5}$	$1.1*10^{-3}$
ENSTGUT00000002323	ENSTGUG00000002234	phenylethanolamine N-methyltransferase	-5.2	$1.0*10^{-4}$	$7.1*10^{-3}$
ENSTGUT00000001273	ENSTGUG00000001218	CAP, adenylate cyclase-associated protein 1	-5.2	$4.0*10^{-20}$	$3.3*10^{-17}$
ENSTGUT00000006208	ENSTGUG00000005964	Uncharacterized protein	-7.6	$3.8*10^{-5}$	$3.1*10^{-3}$

Supplementary Table 5.

Gene identification details for the most highly differentiated genes ($F_{ST} > 0.15$) between the two house finch populations. Given is the F_{ST} - value together with the transcript and gene ID numbers for zebra finch orthologs and a description of the function.

F_{ST}	ZF transcript ID	ZF gene ID	Description
0.33	ENSTGUT00000002033	ENSTGUG00000001957	retinoic acid induced 14
0.33	ENSTGUT00000007759	ENSTGUG00000007429	S-antigen; retina and pineal gland (arrestin)
0.31	ENSTGUT00000018845	ENSTGUG00000018178	Uncharacterized protein
0.28	ENSTGUT00000002897	ENSTGUG00000002782	ectodysplasin A2 receptor
0.27	ENSTGUT00000010213	ENSTGUG00000009796	family with sequence similarity 120B
0.27	ENSTGUT00000005070	ENSTGUG00000004851	unc-45 homolog B (C. elegans)
0.25	ENSTGUT00000001784	ENSTGUG00000001716	Uncharacterized protein
0.24	ENSTGUT00000006600	ENSTGUG00000006352	zinc finger protein 462
0.24	ENSTGUT00000002805	ENSTGUG00000002702	ephrin-B1
0.23	ENSTGUT00000012820	ENSTGUG00000012315	four and a half LIM domains 5
0.23	ENSTGUT00000010930	ENSTGUG00000010458	5-methyltetrahydrofolate-homocysteine methyltransferase
0.23	ENSTGUT00000011143	ENSTGUG00000010682	SAM and SH3 domain containing 1
0.23	ENSTGUT00000005928	ENSTGUG00000005690	ankyrin repeat and BTB (POZ) domain containing 2
0.22	ENSTGUT00000003052	ENSTGUG00000002911	myeloid/lymphoid or mixed-lineage leukemia 5
0.22	ENSTGUT00000004826	ENSTGUG00000004645	hyaluronoglucosaminidase 2
0.22	ENSTGUT00000010402	ENSTGUG00000009965	component of oligomeric golgi complex 2
0.21	ENSTGUT00000009915	ENSTGUG00000009503	toll interacting protein
0.21	ENSTGUT00000005842	ENSTGUG00000005550	dedicator of cytokinesis 8
0.21	ENSTGUT00000011252	ENSTGUG00000010801	receptor accessory protein 1
0.21	ENSTGUT00000008227	ENSTGUG00000007908	Ras association (RalGDS/AF-6) domain family (N-terminal) member 9
0.21	ENSTGUT00000005518	ENSTGUG00000005314	neuropeptide Y receptor Y2
0.21	ENSTGUT00000009250	ENSTGUG00000008866	RAP1 GTPase activating protein 2

0.21	ENSTGUT00000012516	ENSTGUG00000012013	TRAF3 interacting protein 2
0.21	ENSTGUT00000005492	ENSTGUG00000005280	5'-nucleotidase, cytosolic III
0.20	ENSTGUT00000006153	ENSTGUG00000005913	RAB3A interacting protein (rabin3)-like 1
0.20	ENSTGUT00000011305	ENSTGUG00000010845	microtubule-associated protein 7
0.20	ENSTGUT00000005508	ENSTGUG00000005289	transcription factor EC
0.20	ENSTGUT00000006009	ENSTGUG00000005767	elongation of very long chain fatty acids protein 2
0.19	ENSTGUT00000007323	ENSTGUG00000007041	Uncharacterized protein
0.19	ENSTGUT00000011326	ENSTGUG00000010869	Uncharacterized protein
0.19	ENSTGUT00000012349	ENSTGUG00000011851	family with sequence similarity 118, member A
0.19	ENSTGUT00000019080	ENSTGUG00000018324	Uncharacterized protein
0.18	ENSTGUT00000010544	ENSTGUG00000010105	TBC1 domain family, member 14
0.18	ENSTGUT00000002769	ENSTGUG00000002666	pyrimidinergic receptor P2Y, G-protein coupled, 4
0.18	ENSTGUT00000000566	ENSTGUG00000000543	NOP16 nucleolar protein homolog
0.18	ENSTGUT00000005263	ENSTGUG00000005014	X-ray repair complementing defective repair
0.18	ENSTGUT00000001746	ENSTGUG00000001674	nucleolar protein family 6
0.18	ENSTGUT00000002180	ENSTGUG00000002069	nucleoporin 155kDa
0.18	ENSTGUT00000007253	ENSTGUG00000006985	Uncharacterized protein
0.17	ENSTGUT00000002533	ENSTGUG00000002443	Uncharacterized protein
0.17	ENSTGUT00000018073	ENSTGUG00000017392	Uncharacterized protein
0.17	ENSTGUT00000013429	ENSTGUG00000012894	kinesin family member 26A
0.17	ENSTGUT00000012125	ENSTGUG00000011628	ectonucleotide pyrophosphatase/phosphodiesterase 1
0.17	ENSTGUT00000012426	ENSTGUG00000011930	zinc finger with UFM1-specific peptidase domain
0.17	ENSTGUT00000016260	ENSTGUG00000015636	Uncharacterized protein
0.17	ENSTGUT00000016042	ENSTGUG00000015425	protease-associated domain containing 1
0.17	ENSTGUT00000002023	ENSTGUG00000001941	cyclin A2
0.17	ENSTGUT00000013410	ENSTGUG00000012877	cysteine and histidine-rich domain (CHORD) containing 1
0.17	ENSTGUT00000013320	ENSTGUG00000012790	Uncharacterized protein
0.17	ENSTGUT00000004772	ENSTGUG00000004594	PRP38 pre-mRNA processing factor 38 (yeast) domain containing B
0.16	ENSTGUT00000009086	ENSTGUG00000008720	lemur tyrosine kinase 2

0.16	ENSTGUT00000002372	ENSTGUG00000002282	BEN domain containing 7
0.16	ENSTGUT00000010681	ENSTGUG00000010236	Uncharacterized protein
0.16	ENSTGUT00000014064	ENSTGUG00000013505	RCSD domain containing 1
0.16	ENSTGUT00000002053	ENSTGUG00000001974	alanine--glyoxylate aminotransferase 2
0.16	ENSTGUT00000003540	ENSTGUG00000003406	chromosome 16 open reading frame 13
0.16	ENSTGUT00000006377	ENSTGUG00000006125	solute carrier family 38, member 2
0.16	ENSTGUT00000004737	ENSTGUG00000004541	Rho GTPase activating protein 40
0.16	ENSTGUT00000012238	ENSTGUG00000011740	branched chain amino-acid transaminase 1, cytosolic
0.16	ENSTGUT00000005878	ENSTGUG00000005644	phosphoglucomutase 5
0.16	ENSTGUT00000011962	ENSTGUG00000011475	Uncharacterized protein
0.16	ENSTGUT00000012440	ENSTGUG00000011943	dermatan sulfate epimerase
0.16	ENSTGUT00000009309	ENSTGUG00000008927	Uncharacterized protein
0.15	ENSTGUT00000002966	ENSTGUG00000002847	tripartite motif containing 23
0.15	ENSTGUT00000008743	ENSTGUG00000008403	consortin, connexin sorting protein
0.15	ENSTGUT00000006736	ENSTGUG00000006483	additional sex combs like 1
0.15	ENSTGUT00000003939	ENSTGUG00000003782	tet methylcytosine dioxygenase
0.15	ENSTGUT00000008270	ENSTGUG00000007948	Uncharacterized protein
0.15	ENSTGUT00000002100	ENSTGUG00000002018	fibroblast growth factor 2
0.15	ENSTGUT00000008234	ENSTGUG00000007880	alanyl-tRNA synthetase 2, mitochondrial
0.15	ENSTGUT00000003931	ENSTGUG00000003766	Uncharacterized protein
0.15	ENSTGUT00000002263	ENSTGUG00000002170	receptor (TNFRSF)-interacting serine-threonine kinase 1
0.15	ENSTGUT00000010957	ENSTGUG00000010507	family with sequence similarity 193, member A
0.15	ENSTGUT00000009035	ENSTGUG00000008671	procollagen C-endopeptidase enhancer 2
0.15	ENSTGUT00000012791	ENSTGUG00000012285	ubiquitin specific peptidase 45
0.15	ENSTGUT00000017693	ENSTGUG00000017026	KIAA1161
